# Feeding-induced hepatokine, Manf, ameliorates diet-induced obesity by promoting adipose browning via p38 MAPK pathway

**DOI:** 10.1084/jem.20201203

**Published:** 2021-04-15

**Authors:** Tong Wu, Qinhui Liu, Yanping Li, Hong Li, Lei Chen, Xuping Yang, Qin Tang, Shiyun Pu, Jiangying Kuang, Rui Li, Ya Huang, Jinhang Zhang, Zijing Zhang, Jian Zhou, Cuiyuan Huang, Guorong Zhang, Yingnan Zhao, Min Zou, Wei Jiang, Li Mo, Jinhan He

**Affiliations:** 1 Department of Pharmacy, National Clinical Research Center for Geriatrics, West China Hospital, Sichuan University, Chengdu, China; 2 Laboratory of Clinical Pharmacy and Adverse Drug Reaction, National Clinical Research Center for Geriatrics, West China Hospital, Sichuan University, Chengdu, China; 3 Molecular Medicine Research Center, West China Hospital of Sichuan University, Chengdu, China; 4 Center of Gerontology and Geriatrics, National Clinical Research Center for Geriatrics, West China Hospital, Sichuan University, Chengdu, China

## Abstract

Activating beige adipocytes in white adipose tissue (WAT) to increase energy expenditure is a promising strategy to combat obesity. We identified that mesencephalic astrocyte–derived neurotrophic factor (Manf) is a feeding-induced hepatokine. Liver-specific Manf overexpression protected mice against high-fat diet–induced obesity and promoted browning of inguinal subcutaneous WAT (iWAT). Manf overexpression in liver was also associated with decreased adipose inflammation and improved insulin sensitivity and hepatic steatosis. Mechanistically, Manf could directly promote browning of white adipocytes via the p38 MAPK pathway. Blockade of p38 MAPK abolished Manf-induced browning. Consistently, liver-specific Manf knockout mice showed impaired iWAT browning and exacerbated diet-induced obesity, insulin resistance, and hepatic steatosis. Recombinant Manf reduced obesity and improved insulin resistance in both diet-induced and genetic obese mouse models. Finally, we showed that circulating Manf level was positively correlated with BMI in humans. This study reveals the crucial role of Manf in regulating thermogenesis in adipose tissue, representing a potential therapeutic target for obesity and related metabolic disorders.

## Teaser

This article shows Manf, a feeding-induced hepatokine, can directly promote browning of white adipose tissue via the p38 MAPK pathway and consequently protect mice against high-fat diet–induced obesity.

## Graphical Abstract

**Figure:**
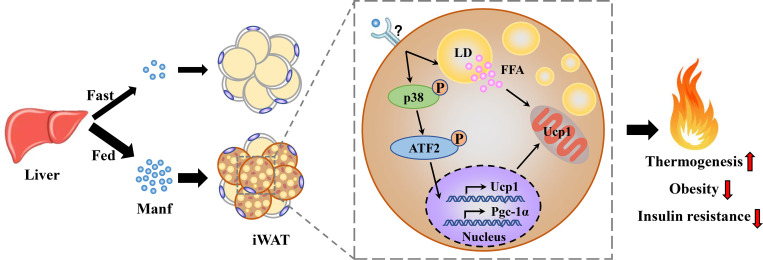

## Introduction

Obesity is a risk factor for the occurrence and development of many metabolic diseases such as diabetes and cardiovascular diseases (González-Muniesa et al., 2017). Obesity is the result of an imbalance between energy intake and energy expenditure. Adipose tissues play a crucial role in sustaining whole-body energy homoeostasis.

There are two types of adipose tissues in mammals, white adipose tissue (WAT) and brown adipose tissue (BAT; Wang and Seale, 2016). WAT stores excess energy as triglycerides. In contrast, BAT dissipates chemical energy as heat through nonshivering thermogenesis mainly mediated by uncoupling protein 1 (Ucp1) in mitochondria. Recent studies have uncovered another special type of adipose tissue called beige (or brite) fat within WAT. Beige fat appears in response to hormonal or environmental stimulation (especially in inguinal subcutaneous WAT [iWAT]), which, showing biochemical and morphological features similar to classical BAT, highly expresses Ucp1 and performs uncoupled mitochondrial respiration (Giralt and Villarroya, 2013; Shabalina et al., 2013). The accumulation of these beige adipocytes is referred to as “browning.” Browning of WAT was first found by Young et al. in 1984 in female BALB/c mice exposed to cold acclimation for 4 wk (Young et al., 1984), but it has not been observed in humans yet. So far, scientists have confirmed that browning of WAT could lead to increased thermogenesis and is critical to combat obesity and its related metabolic disorders (Vargas-Castillo et al., 2017).

Feeding can increase thermogenesis in humans and rodents. A study of 17 women and 20 men found up-regulated energy expenditure after a meal (Westerterp, 2004). In addition, U Din et al (2018) found that whole-body and BAT thermogenesis were induced after a meal. Rothwell and Stock (1979) demonstrated thermogenesis induced by feeding in rats: overfed rats showed a 100% increase in energy expenditure. In mice, nonshivering thermogenesis was significantly reduced with fasting and could be recovered by refeeding (Trayhurn and Jennings, 1988). The mechanism of feeding-induced thermogenesis is less studied. β-Adrenergic receptor, neuropeptide FF receptor-2, and apolipoprotein A-IV have been proposed to participate in feeding-induced thermogenesis; KO of these genes caused obesity by impairing feeding-induced thermogenesis (Bachman et al., 2002; Bai et al., 2018; Pence et al., 2019). In adipose tissue, inhibition of creatine biosynthesis decreased diet-induced thermogenesis and drove obesity (Kazak et al., 2017). Feeding also increased the adipose expression of fibroblast growth factor 21 (Fgf21; Fisher et al., 2012), which promoted browning in an autocrine/paracrine manner.

Liver plays an important role in energy metabolism. Feeding could induce the liver to release hormones (referred as hepatokines) that act on other tissues to regulate glucose and lipid metabolism. Feeding is known to induce the circulating level of proprotein convertase subtilisin/kexin type 9 (Pcsk9) and angiopoietin-like protein 8 (Angptl8), which regulate cholesterol and triglyceride metabolism in lipoproteins (Persson et al., 2010; Quagliarini et al., 2012). Adropin is also a secreted factor from the liver (Kumar et al., 2008). Serum adropin levels were lower after fasting in mice and could protect mice against adiposity, insulin resistance, and dyslipidemia (Ganesh Kumar et al., 2012). However, whether the liver could respond to feeding and secrete hepatokines to regulate thermogenesis remains unclear.

Mesencephalic astrocyte-derived neurotrophic factor (Manf) is a secreted protein containing a secretory signal peptide sequence of 21 amino acids at the N terminus, a mature protein sequence of 158 amino acids (Mizobuchi et al., 2007). Manf was retained in cells and is secreted by ER stress (Glembotski et al., 2012). Recent reports indicated that Manf may be involved in metabolic homeostasis (Imran et al., 2017; Sousa-Victor et al., 2019). Here, we identified Manf as a hepatokine induced by refeeding. Liver-specific overexpression of Manf reduced diet-induced obesity by increasing energy expenditure via promoting the browning of white adipocytes. In contrast, liver-specific Manf ablation exacerbated diet-induced obesity accompanied by impaired thermogenesis. Mechanistically, Manf could directly promote the browning of adipocytes via the p38 MAPK pathway. Furthermore, recombinant Manf ameliorated obesity-related metabolic disorders by increasing thermogenesis. Serum Manf level was positively correlated with body mass index (BMI) in humans. Our study suggests that feeding-induced Manf is involved in energy homeostasis and may be a therapeutic target of metabolic disease.

## Results

### Manf is a hepatokine up-regulated by refeeding

**Figure 1.**
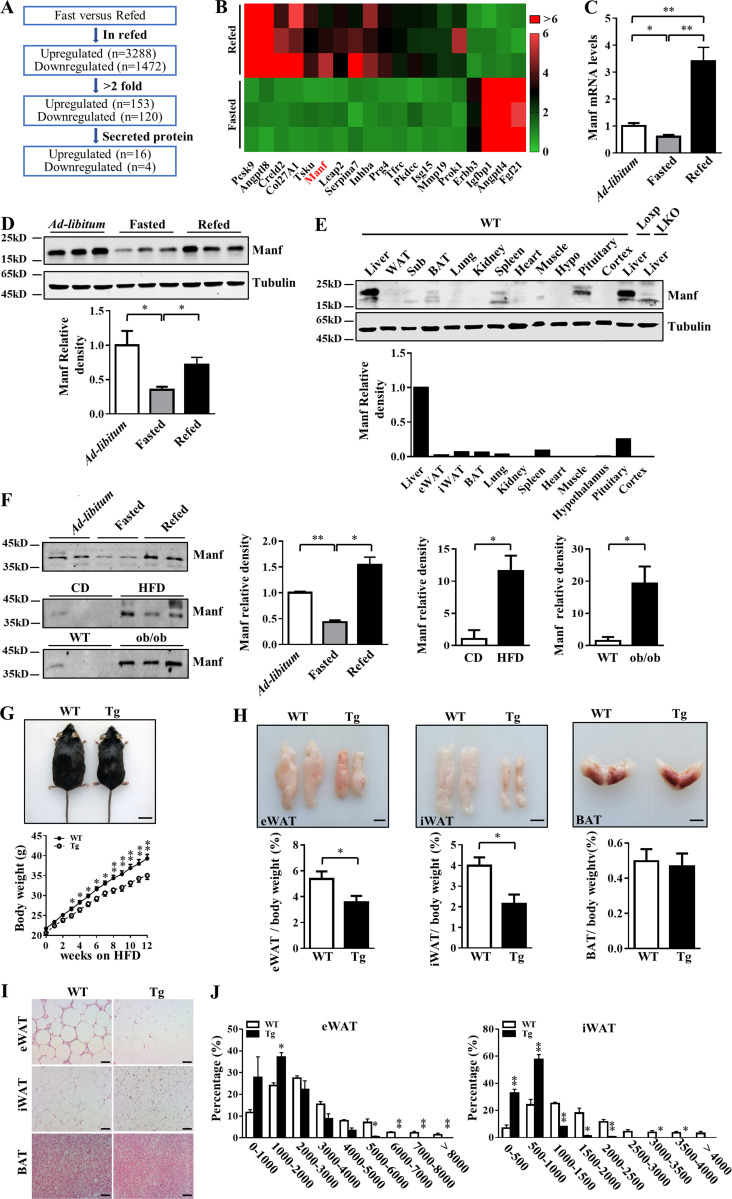
**Hepatic Manf was up-regulated by refeeding and can protect mice from HFD-induced obesity.** **(A and B)** Schematic representation of screening procedure (A) and heat map representation (B) of hepatokines deferentially expressed in 8-wk-old fasting and refeeding (Refed) mice (*n* = 3). **(C)** The mRNA level of Manf in liver from ad libitum, fasting, or refeeding mice (*n* = 4). 8-wk-old male mice were fasted for 24 h with free access to water; refeeding mice were fasted for 20 h and then refed for 4 h. **(D)** The protein levels of Manf in liver from ad libitum, fasting, and refeeding mice. **(E)** Protein levels of Manf in different tissues of 8-wk-old male mice. **(F)** Protein levels of Manf in serum from ad libitum, fasting, or refeeding WT mice, fasted HFD-fed mice, and ob/ob mice (*n* = 3). **(G)** Appearance (top) and growth curve (bottom) of WT (*n* = 22) and Tg (*n* = 24) mice fed with HFD for 12 wk. Scale bar = 2 cm. **(H)** Representative photographs of eWAT, iWAT, and BAT (top) and the ratio of fat depots to body weight (*n* = 6). Scale bar = 1 cm. **(I and J)** H&E staining of adipose tissues (I) and distribution of adipocyte size of eWAT and iWAT (J). Scale bar = 50 µm. Each experiment was independently performed two to three times. All data are mean ± SEM. *, P < 0.05; **, P < 0.01. Hypo, hypothalamus.

**Figure S1.**
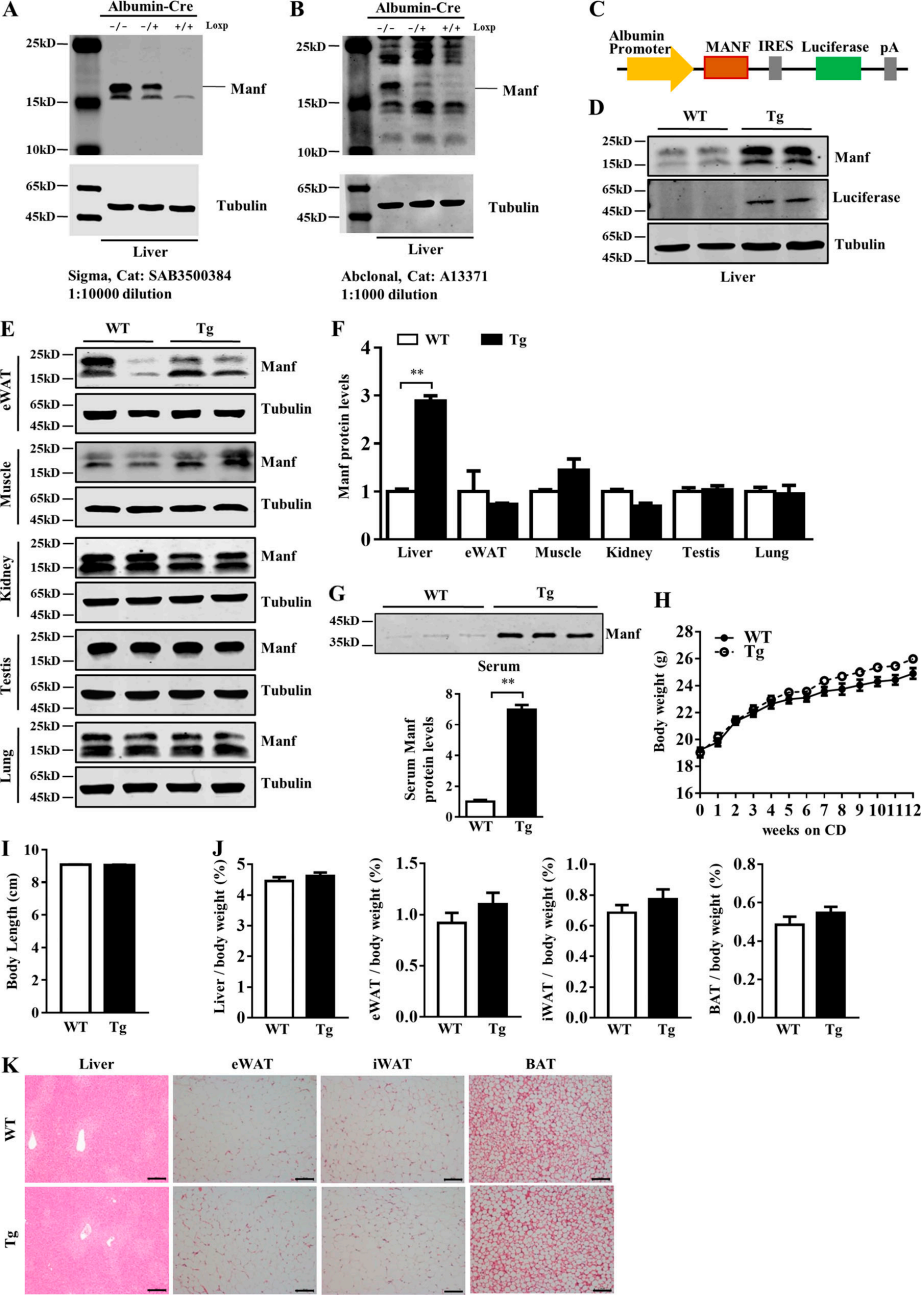
**Tg mice showed similar body weight, body length, and tissue morphology under CD. (A and B)** Validation of anti-Manf antibody in liver samples. Manf band can be detected at ∼18 kD and is nearly absent in LKO mice. **(C)** Schematic showing the strategy for generation of Tg mice. **(D–F)** Protein levels of Manf in liver and other tissues. **(G)** Manf levels in serum (*n* = 3). **(H)** Growth curve of WT (*n* = 11) and Tg (*n* = 8) mice fed with CD for 12 wk. **(I)** Body length of WT and Tg mice (*n* = 6). **(J)** the ratio of tissues weight to body weight (*n* = 6). **(K)** H&E staining of liver, eWAT, iWAT, and BAT. Scale bar = 100 µm. Each experiment was independently performed two to three times. All data are mean ± SEM. **, P < 0.01.

To identify potential feeding-induced hepatokines, we performed RNA sequencing analysis of livers from fasting and refeeding mice. Re-feeding induced a cluster of 153 genes that were up-regulated and 120 genes that were down-regulated more than twofold (P < 0.05; Fig. 1 A). 16 up-regulated and 4 down-regulated genes had a signal peptide for secretion. Many of these secreted proteins have been found to be regulated by fasting or refeeding, including Fgf21 and Pcsk9 (Fig. 1 B; Fisher et al., 2012; Lagace, 2014). We finally focused on Manf because of its high expression in liver and unknown function in energy metabolism. Quantitative PCR (qPCR) analysis confirmed that refeeding greatly increased mRNA levels of *Manf* (Fig. 1 C). Protein levels of Manf were consistently higher after refeeding in liver (Fig. 1 D). The Manf antibodies from different companies were validated (Fig. S1, A and B). To characterize the Manf expression pattern, we analyzed its protein level in different tissues. Consistent with a previous report (Lindholm et al., 2008), Manf was expressed in the brain. Surprisingly, the liver showed the highest expression of Manf (Fig. 1 E). The other tissues such as adipose and muscle showed much lower levels. As a secreted protein, serum Manf levels were higher after refeeding and in the overnutrition mice models (Fig. 1 F). Thus, Manf was a hepatokine regulated by nutritional status and may play a role in obesity and energy homeostasis.

### Liver-specific Manf overexpression reduced high-fat diet (HFD)–induced obesity

To explore the potential role of hepatic-released Manf, we generated liver-specific Manf transgenic (Tg) mice (Fig. S1 C). The expression of Manf was specifically increased in liver of Tg mice as compared with WT mice (Fig. S1, D–F). Serum Manf level was higher in Tg than WT mice (Fig. S1 G).

**Figure S2.**
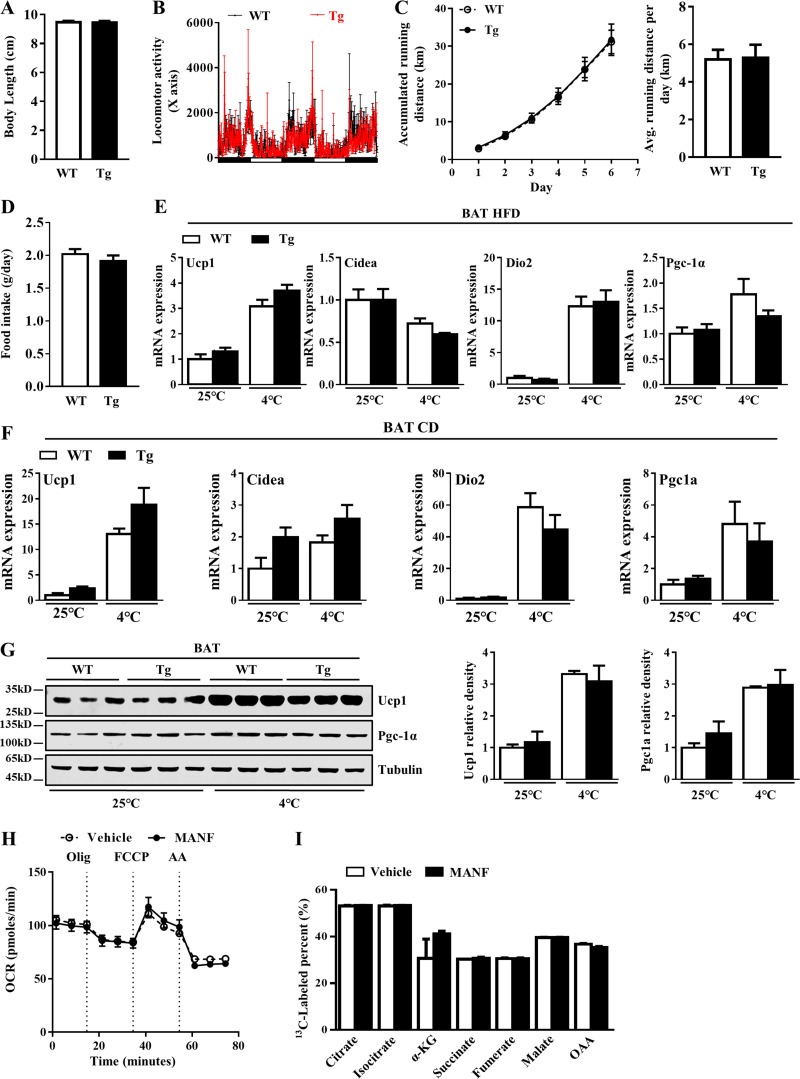
**Manf overexpression did not affect physical activity and the function of BAT. (A)** Body length of mice fed with HFD (*n* = 6). **(B)** Locomotor activity of WT and Tg mice housed in individual metabolic cages (*n* = 5). **(C)** Accumulated (left) and daily average (right) running distance (*n* = 5). **(D)** Food intake of mice fed with HFD (*n* = 6). **(E and F) **qPCR analysis of thermogenic genes in BAT from mice fed with HFD (E) or CD (F) for 12 wk (*n* = 6). **(G)** The protein levels of Ucp1 and Pgc-1α in BAT from mice fed with HFD for 12 wk (*n* = 3). **(H and I) **Oxygen consumption rate (H) and fatty acid oxidation (I) of primary brown adipocytes incubated with or without Manf (*n* = 3). Each experiment was independently performed two to three times. All data are mean ± SEM. Avg., average; OCR, oxygen consumption rate; Olig, oligomycin; FCCP, carbonyl cyanide-4 (trifluoromethoxy) phenylhydrazone, AA, antimycin-A; α-KG, α-ketoglutarate; OAA, oxaloacetate.

WT and Tg mice on a chow diet (CD) showed similar body weight, body length, and tissue morphology (Fig. S1, H–K). However, on an HFD, Tg mice body weight diverged from that of WT mice after 3 wk of challenge; after 12 wk of HFD feeding, Tg mice were visibly smaller and gained less body weight than WT mice (Fig. 1 G). However, body length was similar between WT and Tg mice (Fig. S2 A). To determine the cause of the decreased body weight in Tg mice, we weighed adipose tissues. Epididymal WAT (eWAT) and iWAT from Tg mice were smaller and weighed less than those from WT mice (Fig. 1 H). The lower adipose weight was likely due to decreased adipocyte size in Tg mice (Fig. 1, I and J). The weight of BAT was similar between WT and Tg mice (Fig. 1 H).

### Manf overexpression increased whole-body energy expenditure and iWAT browning

**Figure 2.**
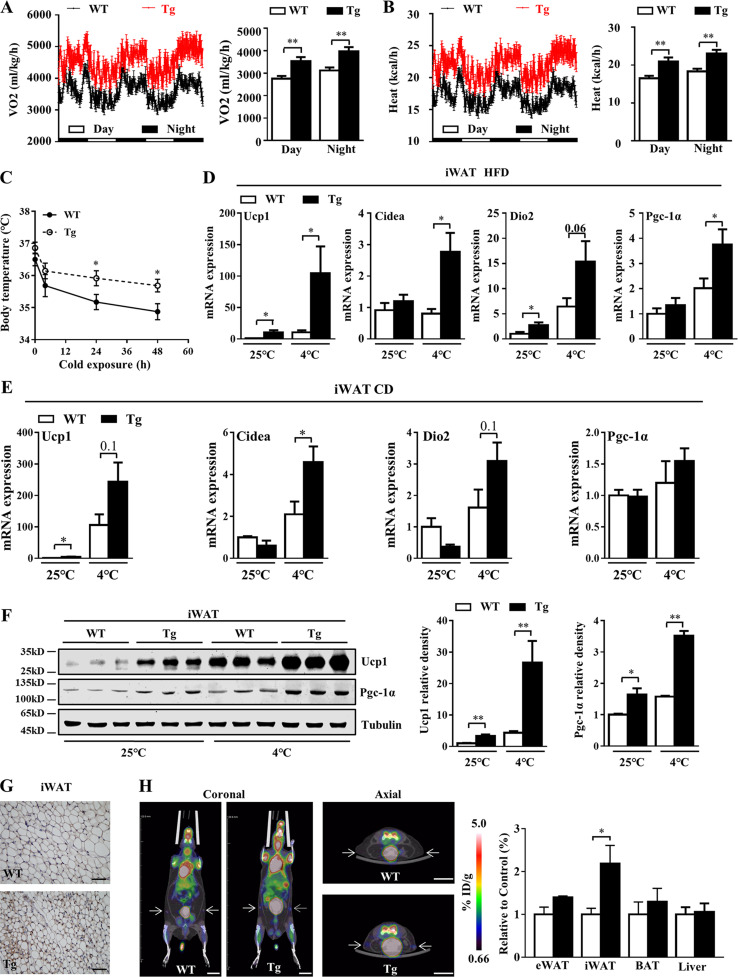
**Manf overexpression increased whole-body energy expenditure and iWAT browning. (A and B)** Oxygen consumption (A) and whole-body energy expenditure (B) of WT and Tg mice fed an HFD for 12 wk (*n* = 5). **(C)** Rectal temperature of 12-wk-HFD WT and Tg mice during cold exposure (4°C; *n* = 7). **(D and E)** qPCR analysis of genes involved in thermogenesis in iWAT from mice fed with HFD****(D) or CD (E) for 12 wk (*n* = 4–8). **(F)** Protein levels of Ucp1 and Pgc-1α in iWAT from mice fed a CD for 12 wk (*n* = 3). **(G)** Representative images of Ucp1 IHC. Scale bar = 50 µm. **(H)** Representative [^18^F]-FDG PET/CT images of WT and Tg mice after 12 wk of HFD, and relative [^18^F]-FDG uptake in adipose tissues and liver (*n* = 4). White bars represent WT mice, and black bars represent Tg mice. White arrows indicate the area of iWAT. Scale bar = 1 cm. Each experiment was independently performed two to three times. All data are mean ± SEM. *, P < 0.05; **, P < 0.01.

To understand the potential mechanisms of Manf overexpression–reduced obesity, we monitored energy intake and energy expenditure. The oxygen consumption was significantly higher in Tg mice during both light and dark cycles (Fig. 2 A). Energy expenditure was consistently increased in Tg mice (Fig. 2 B). In agreement with increased energy expenditure, Tg mice maintained higher body temperature when exposed to 4°C (Fig. 2 C). Physical activity and voluntary activity were also analyzed using metabolic cages and running wheels. Tg mice showed no changes in locomotor activity (Fig. S2 B) and voluntary running distance (Fig. S2 C) as compared with WT mice. Moreover, food intake was comparable between WT and Tg mice (Fig. S2 D).

Because BAT plays an important role in the regulation of body temperature, we analyzed the expression of thermogenic genes in BAT. However, the expression of Ucp1 and other thermogenic genes including cell death–inducing DFFA-like effector a (*Cidea*), deiodinase 2 (*Dio2*), and *Pgc-1α* did not change in BAT of Tg mice as compared with WT mice (Fig. S2, E–G). Consistently, in primary BAT adipocytes, recombinant Manf did not affect oxygen consumption (Fig. S2 H) and fatty acid oxidation (Fig. S2 I) activity. These results suggest that elevated thermogenesis in Tg mice was not due to the activation of BAT.

Browning of WAT has been shown to contribute to adaptive thermogenesis (Bartelt and Heeren, 2014). We next examined the metabolic consequence of liver-specific Manf overexpression in WAT. In iWAT, Manf overexpression significantly increased the mRNA expression of Ucp1 and other thermogenic genes in Tg mice as compared with WT mice under both HFD (Fig. 2 D) and CD (Fig. 2 E) conditions. Western blot (WB) analysis further confirmed the increased protein level of Ucp1 and Pgc-1α in Tg mice at both 25°C and 4°C (Fig. 2 F). Furthermore, immunohistochemistry (IHC) staining showed higher Ucp1 expression in iWAT of Tg than WT mice (Fig. 2 G). Positron emission tomography (PET)/computed tomography (CT) with [^18^F]-fluorodeoxyglucose ([^18^F]-FDG) can be used to observe activated beige adipocytes (Park et al., 2015). As expected, PET/CT clearly showed a marked increase in [^18^F]-FDG uptake in iWAT but not BAT of Tg mice versus WT mice (Fig. 2 H). These data indicate that the lower body weight seen in Tg mice likely resulted from increased adaptive thermogenesis due to iWAT browning.

### Manf directly stimulated browning via the p38 MAPK pathway

**Figure 3.**
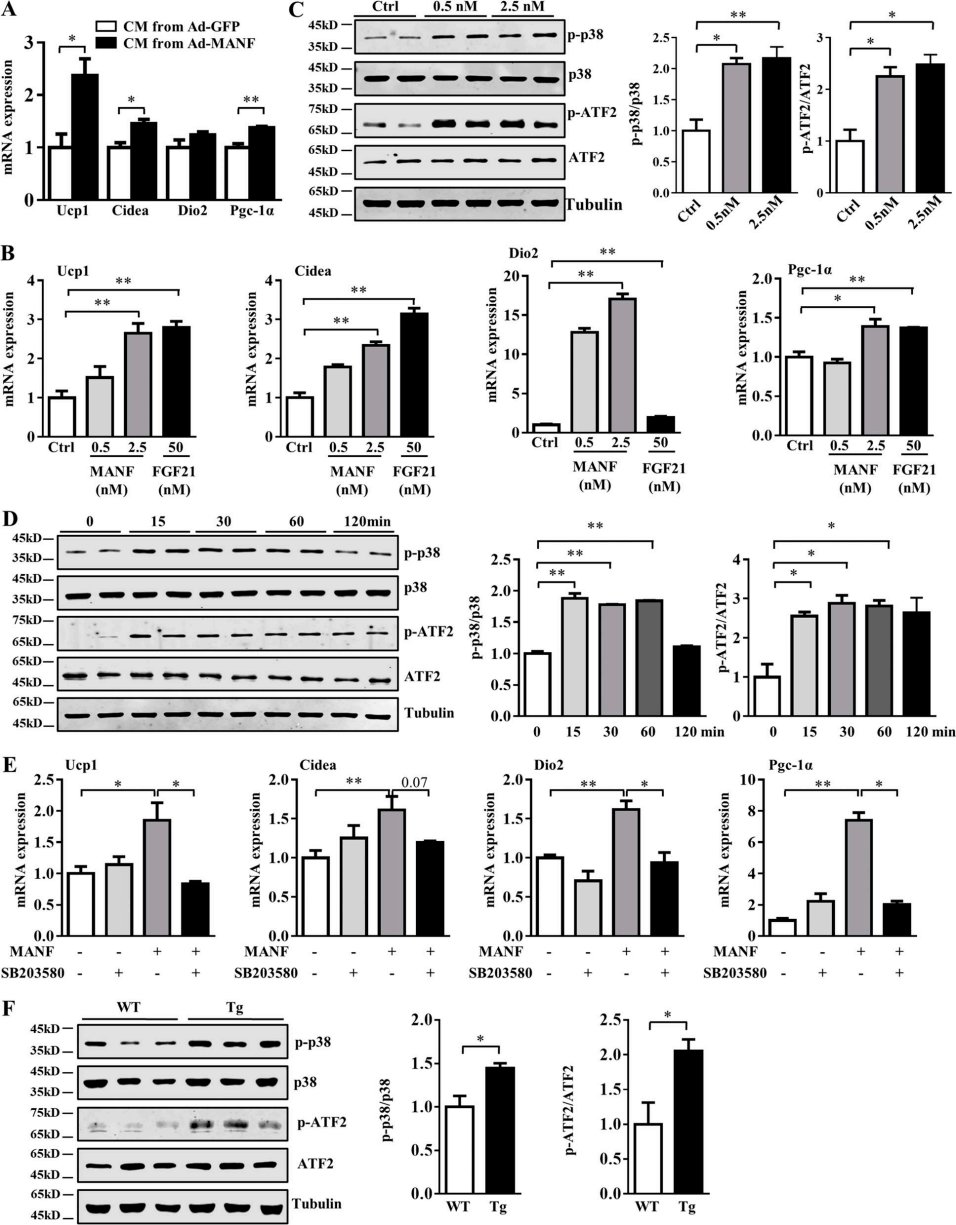
**Manf stimulated browning via p38 MAPK signaling. (A)** qPCR analysis of thermogenic genes in primary iWAT incubated with CM from primary hepatocytes infected with Ad-GFP or Ad-Manf for 48 h (*n* = 3). **(B)** Thermogenic gene expression in primary iWAT treated with recombinant Manf and Fgf21 (*n* = 3). **(C)** Phosphorylated of p38 and ATF2 in primary adipocyte from iWAT treated with different concentration of Manf for 30 min. **(D)** Phosphorylated and total p38 and ATF2 in primary adipocyte from iWAT incubated with 2.5 nM Manf for different periods of time. **(E)** Primary adipocytes were pretreated with SB203580 (10 µM) for 2 h, then Manf (2.5 nM) for an additional 48 h. mRNA levels of thermogenic genes were detected by qPCR (*n* = 3). **(F)** Phosphorylated and total p38 and ATF2 protein levels in iWAT from WT and Tg mice fed an HFD for 12 wk (*n* = 3). Each experiment was independently performed two to three times. All data are mean ± SEM. *, P < 0.05; **, P < 0.01. Ctrl, control.

**Figure S3.**
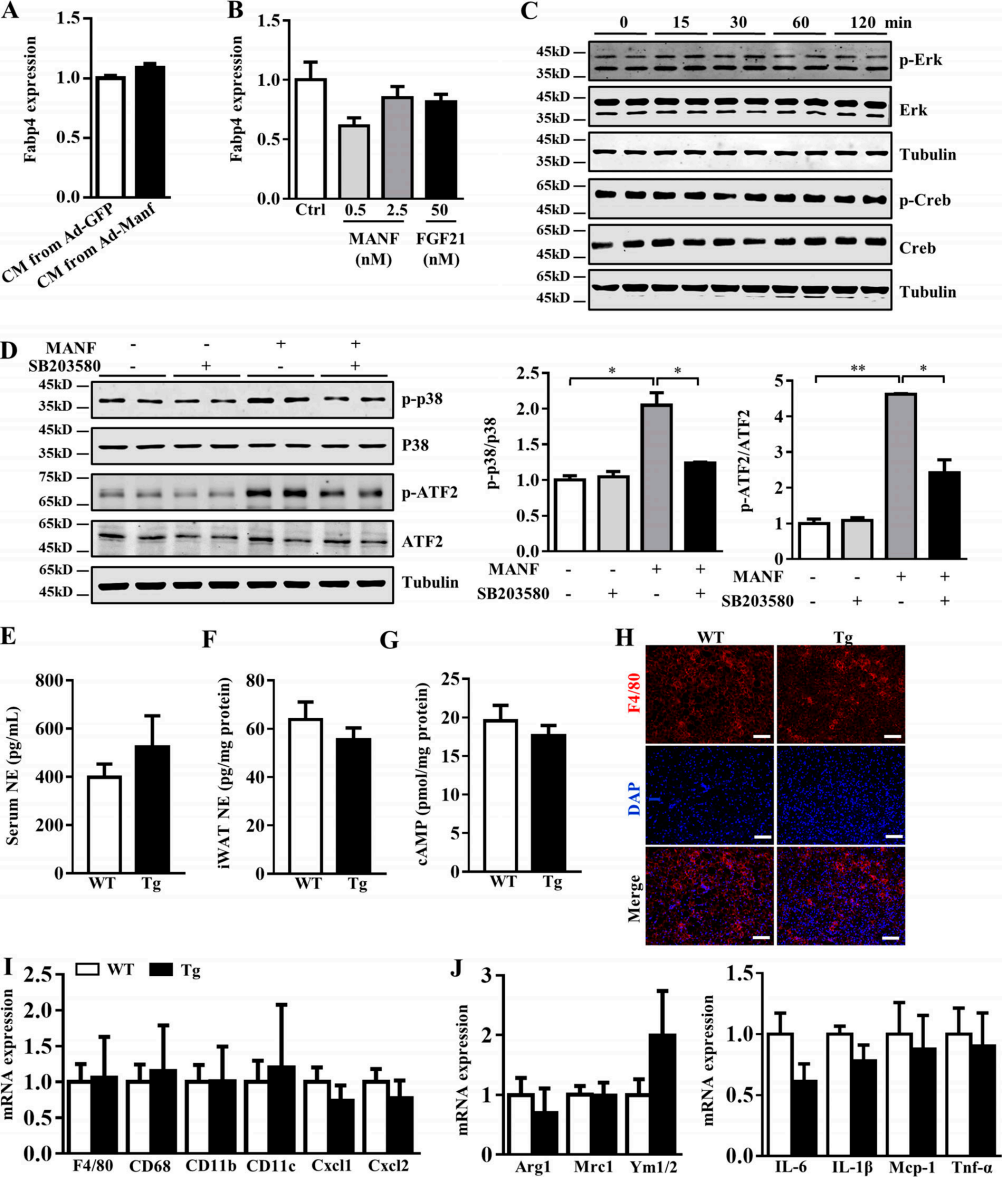
**Manf did not affect cAMP, Erk, and Creb signaling pathway. (A)** The expression of Fabp4 in primary adipocytes from iWAT incubated with CM collected from primary hepatocytes infected with Ad-GFP or Ad-Manf (*n* = 6). **(B)** mRNA levels of Fabp4 in primary adipocytes from iWAT treated with recombinant Manf (*n* = 3). **(C)** Phosphorylated and total Erk and Creb in primary iWAT treated with recombinant Manf (2.5 nM; *n* = 2). **(D)** The levels of p-p38 in primary adipocytes pretreated with p38 MAPK inhibitor SB023580 (SB; 10 µM) for 2 h followed by Manf (2.5 nM) treatment for 30 min (*n* = 2). **(E and F) **Serum and iWAT norepinephrine (NE) levels in WT and Tg mice on an HFD (*n* = 5 or 6). **(G)** cAMP levels of iWAT from WT and Tg mice on an HFD (*n* = 6). **(H) **Immunofluorescent staining of F4/80 in BAT from HFD-fed mice. **(I and J) **mRNA levels of markers of M1 and M2 macrophages and inflammatory genes in BAT from HFD-fed mice. Each experiment was independently performed two to three times. All data are mean ± SEM. *, P < 0.05; **, P < 0.01. Ctrl, control.

To determine whether iWAT browning was the result of hepatocyte-derived Manf stimulation, we collected the conditioned medium (CM) from primary hepatocytes infected with adenovirus (Ad)-Manf or Ad-GFP and incubated adipocytes with this CM. Consistently, adipocytes incubated with CM from Ad-Manf–infected hepatocytes showed higher mRNA levels of *Ucp1*, *Cidea*, and *Pgc-1α* (Fig. 3 A). CM had little effect on the differentiation of preadipocytes as indicated by the similar mRNA level of *Fabp4*, a well-established marker of adipocyte differentiation (Fig. S3 A). To directly test the effect of Manf on browning, we treated primary differentiated adipocytes with 0.5–2.5 nM recombinant Manf. Fgf21, a well-known browning inducer, was used as a positive control (Fisher et al., 2012). Recombinant Manf could directly increase the expression of Ucp1 and other thermogenic genes (Fig. 3 B and Fig. S3 B), which suggests that Manf could directly promote adipocyte browning. Thus, we confirmed that Manf could indeed promote the browning of primary adipocytes from iWAT.

To understand the mechanism of Manf-induced browning, we analyzed signaling pathways known to mediate browning. Phosphorylation of p38 MAPK, Creb, and Erk was examined after Manf treatment of primary differentiated adipocytes. The phosphorylation of p38 (p-p38) was increased in a concentration-dependent manner (Fig. 3 C). Moreover, p-p38 was significantly increased at 15 min after the addition of Manf and decreased at 120 min (Fig. 3 D). p38 MAPK further phosphorylates and activates activating transcription factor 2 (ATF2), which induce the transcription of Ucp1 and other thermogenic genes (Harms and Seale, 2013). We further detected the protein levels of phosphorylated and total ATF2. The phosphorylation of ATF2 was higher in Manf-treated adipocytes (Fig. 3, C and D). In contrast, the phosphorylation of Creb and Erk pathways was not changed (Fig. S3 C). To further confirm the involvement of the p38 MAPK pathway in Manf-induced browning, primary adipocytes were pretreated with SB203580, a well-known inhibitor of p38α and p38β. As expected, Manf-induced p-p38 and ATF2 was suppressed by SB203580 (Fig. S3 D). Blockade of the p38 MAPK pathway greatly abolished Manf-increased expression of Ucp1, Dio2, and Pgc-1α (Fig. 3 E). Consistent with the result from in vitro, the p-p38 and p-ATF2 were also significantly increased in iWAT of Tg mice (Fig. 3 F).

The sympathetic nervous system (SNS) plays a key role in browning of adipose tissue (Bordicchia et al., 2012). To determine whether Manf-induced thermogenesis is mediated by SNS–β-adrenoreceptor (β-AR)–cAMP pathway, we analyzed norepinephrine levels in serum and iWAT. No difference was observed (Fig. S3, E and F). cAMP levels were also measured in iWAT, and they were similar between WT and Tg mice (Fig. S3 G). Thus, hepatocyte-secreted Manf could directly promote the browning of adipocytes by activating the p38 MAPK pathway, but did not affect traditional SNS–β-AR–cAMP pathway.

### Manf overexpression increased lipolysis and ameliorated HFD-induced inflammation in adipose tissue

**Figure 4.**
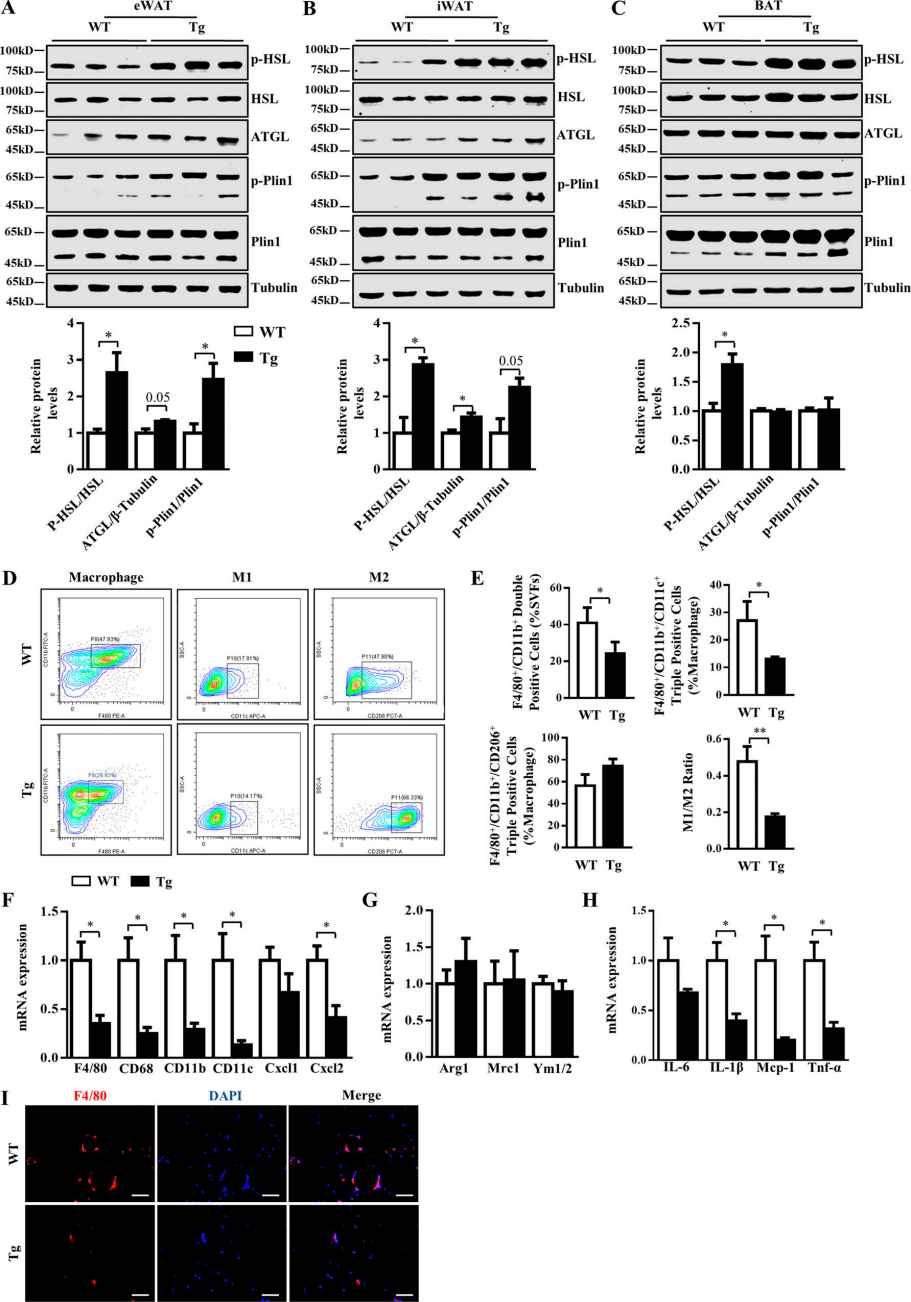
**Manf overexpression increased lipolysis and inhibited HFD-induced inflammation in adipose tissue. (A–C)** Protein levels of key lipolytic proteins in eWAT (A), iWAT (B), and BAT (C) from WT and Tg mice fed an HFD. **(D)** FACS analysis of macrophages in eWAT of WT and Tg mice fed an HFD for 12 wk. **(E)** Percentage of macrophages, M1 and M2 macrophages, and ratio of M1 to M2 (*n* = 3 experiments). **(F–H)** mRNA levels of markers of M1 and M2 macrophages and inflammatory genes in eWAT from HFD-fed mice. **(I)** Immunofluorescent staining of F4/80 in eWAT from HFD-fed mice (*n* = 4–5). Samples were stained with DAPI (blue) or F4/80 (red). Scale bar = 50 µm. Each experiment was independently performed two to three times. All data are mean ± SEM. *, P < 0.05; **, P < 0.01. AUC, area under the curve.

Lipolysis is essential for adipose tissue thermogenesis because the fatty acids released from lipid mobilization serve as both obligatory activators for Ucp1 and metabolic substrates fueling thermogenic respiration (Li et al., 2014). We next assessed the key lipolytic proteins in eWAT, iWAT, and BAT. Hormone-sensitive lipase (HSL) and adipose triglyceride lipase (ATGL) are rate-limiting lipolytic enzymes (Schweiger et al., 2006). The phosphorylation of HSL and the expression of ATGL were significantly increased in both eWAT and iWAT of Tg mice versus WT mice (Fig. 4, A and B). Total and phosphorylated HSLs were up-regulated in BAT of Tg mice (Fig. 4 C). The phosphorylation of perilipin-1 (Plin1) can alleviate the barrier function of this protein and promote its active participation in the lipolytic process (Greenberg et al., 1991). The phosphorylation of Plin1 was significantly increased in both eWAT and iWAT of Tg mice (Fig. 4, A and B). Total and phosphorylated Plin1 was also higher in BAT of Tg mice (Fig. 4 C). These results suggest that increased lipolysis may coordinate with adaptive thermogenesis to promote energy expenditure.

Obesity is accompanied by chronic inflammation in adipose tissues. Adipose tissue inflammation involves the accumulation of adipose tissue macrophages (ATMs; Lumeng et al., 2007). ATMs can be defined by their activation state as classically activated macrophages (M1 macrophages), which secrete proinflammatory cytokines, or alternatively activated macrophages (M2 macrophages), which secrete anti-inflammatory cytokines (Lumeng et al., 2007). We performed flow cytometry to analyze ATMs in eWAT. The total number of F4/80^+^/CD11b^+^ double-positive ATMs was less in eWAT from Tg than WT mice (Fig. 4 D). Manf overexpression also caused a significant decrease in the number of F4/80^+^/CD11b^+^/CD11c^+^ triple-positive M1 microphages (Fig. 4 E). The number of CD206^+^/CD11b^+^/F4/80^+^ triple-positive M2 microphages did not change (Fig. 4 E). As a result, the ratio of M1/M2 was significantly decreased in eWAT of Tg mice as compared with WT mice (Fig. 4 E). Consistently, the expression of markers of M1 macrophages was greatly suppressed in Tg mice (Fig. 4 F), with no change in expression of markers of M2 macrophages (Fig. 4 G). As a result, the expression of inflammatory genes such as *IL-1β*, monocyte chemoattractant protein 1 (*Mcp-1*), and *Tnf-α* was decreased in eWAT of Tg mice versus WT mice (Fig. 4 H). Immunochemistry also revealed decreased staining for F4/80 in eWAT of Tg mice (Fig. 4 I). We also measured the expression of these genes in BAT using immunofluorescence (IF) and qPCR, and no difference was observed (Fig. S3, I and J). Therefore, Manf protected mice against inflammation by inhibiting the polarization of macrophages to M1 in eWAT.

### Manf overexpression improved insulin resistance and hepatic steatosis in HFD-fed mice

**Figure 5.**
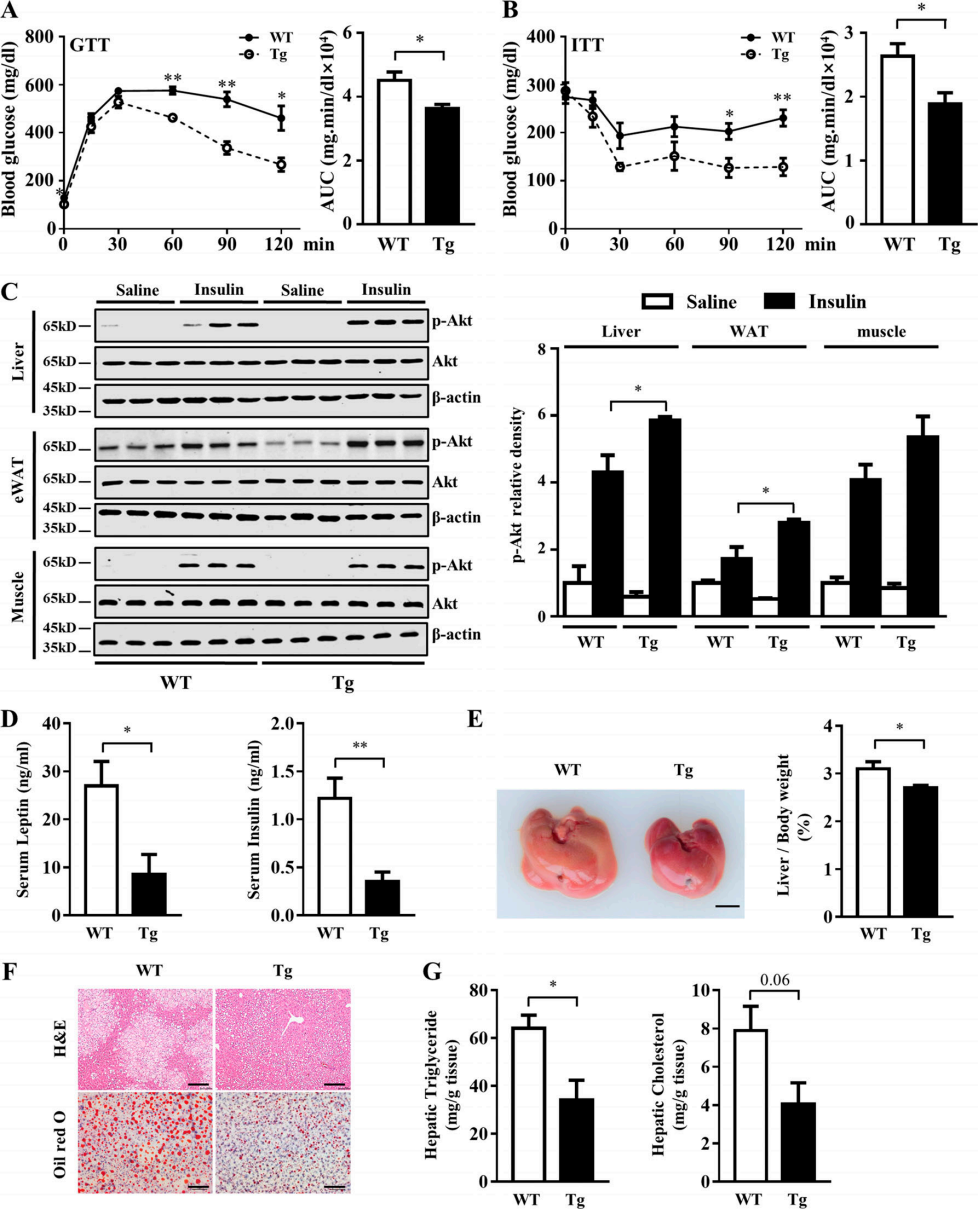
**Manf overexpression alleviated HFD-induced insulin resistance and hepatic steatosis.** WT and Tg mice were fed an HFD for 12 wk. **(A and B)** Blood glucose concentrations during GTT (1 g/kg; A) and ITT (1.5 U/kg; B) in WT and Tg mice (*n* = 6). **(C)** Protein levels of phosphorylated and total Akt in liver, eWAT, and muscle after insulin injection. HFD-fed mice received a bolus injection of insulin (1 U/kg) through the portal vein. 5 min later, tissues were harvested for WB analysis. **(D)** Serum leptin and insulin levels in overnight-fasted WT and Tg mice (*n* = 6). **(E)** A representative image of liver (left) and ratio of liver to body weight (right; *n* = 6). Scale bar = 0.5 cm. **(F)** H&E and Oil Red O staining of liver sections. Scale bar = 50 µm. **(G)** Hepatic lipids levels in liver of WT and Tg mice fed an HFD (*n* = 6). Each experiment was independently performed two to three times. All data are mean ± SEM. *, P < 0.05; **, P < 0.01.

Obesity and inflammation in adipose tissue are major reasons for insulin resistance and glucose intolerance (Lee et al., 2011). HFD-fed Tg mice showed increased glucose tolerance and insulin sensitivity (Fig. 5, A and B). In line with improved insulin resistance, phosphorylation of insulin-stimulated protein kinase B (Akt) was enhanced in liver and eWAT of Tg mice fed an HFD (Fig. 5 C). Decreased circulating levels of serum insulin and leptin also supported the improved insulin resistance in Tg mice (Fig. 5 D).

HFD feeding can lead to hepatic steatosis. As compared with WT mice, the liver of Tg mice fed an HFD was smaller, and the ratio of liver weight to body weight was lower (Fig. 5 E). Oil Red O and H&E staining also showed decreased lipid accumulation in liver of Tg mice (Fig. 5 F). Hepatic triglycerides level was significantly decreased in the liver of Tg mice (Fig. 5 G).

### Liver-specific KO of Manf (LKO) promoted HFD-induced obesity

**Figure S4.**
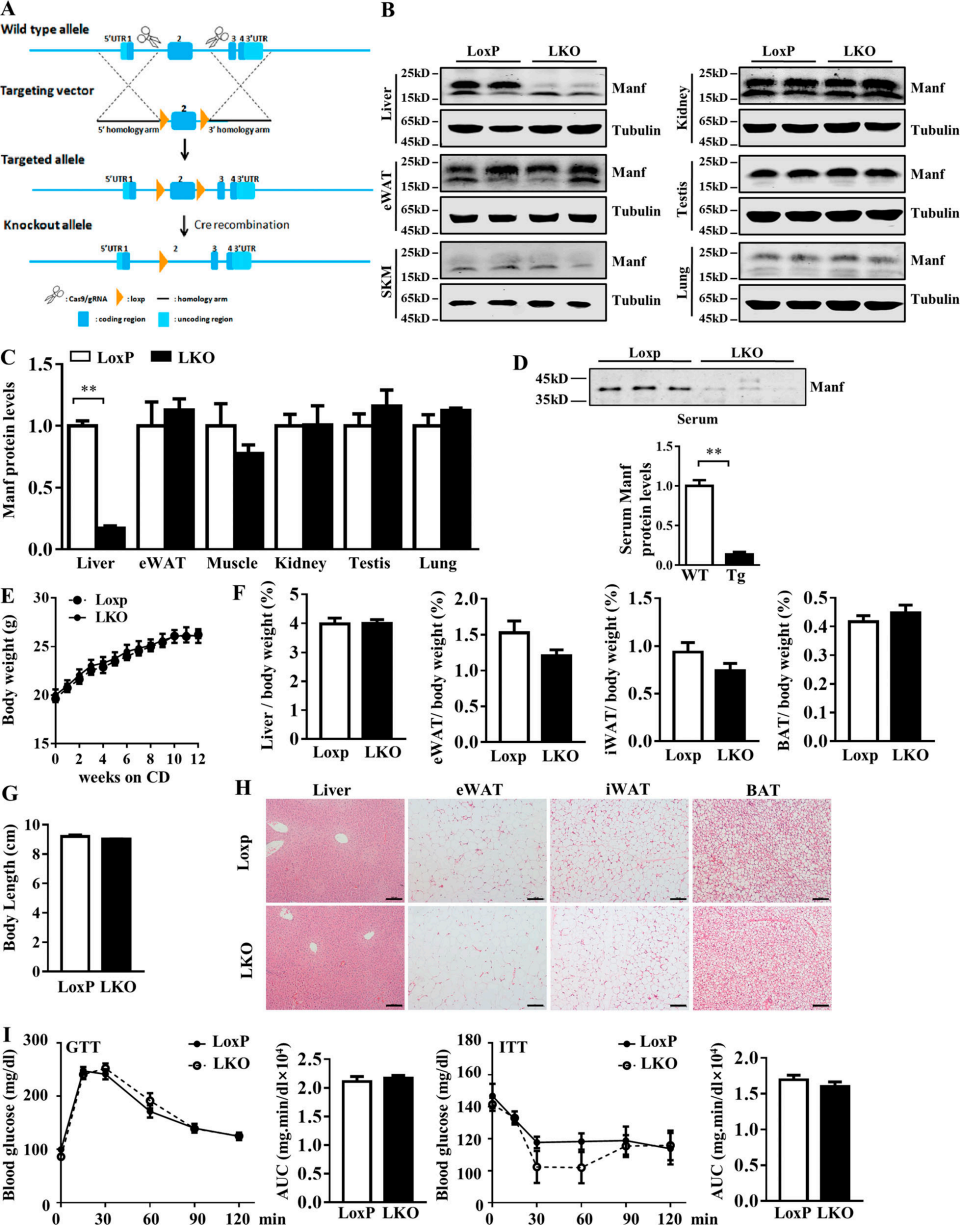
**LKO mice showed similar body weight, tissue morphology, and glucose tolerance under CD. (A)** The schematic diagram of Manf Lxop mice. **(B)** The protein levels of Manf in different tissues of 8-wk-old mice. **(C)** Quantitation of Fig. S4 B. **(D)** The serum levels of Manf in LoxP and LKO mice (*n* = 3). **(E)** Growth curve and body length of LoxP (*n* = 7) and LKO (*n* = 8) mice. **(F)** The ratio of liver, eWAT, iWAT, and BAT weight to body weight of LoxP (*n* = 7) and LKO (*n* = 8) mice. **(G)** Body length of LoxP and LKO mice under CD (*n* = 6). **(H)** H&E staining of liver, eWAT, iWAT, and BAT. **(I)** Blood glucose levels during a GTT (2 g/kg) and an ITT (0.5 U/kg). Scale bar = 100 µm. Each experiment was independently performed two to three times. All data are mean ± SEM. **, P < 0.01. AUC, area under the curve; gRNA, guide RNA; UTR, untranslated region; SKM, skeletal muscle.

**Figure 6.**
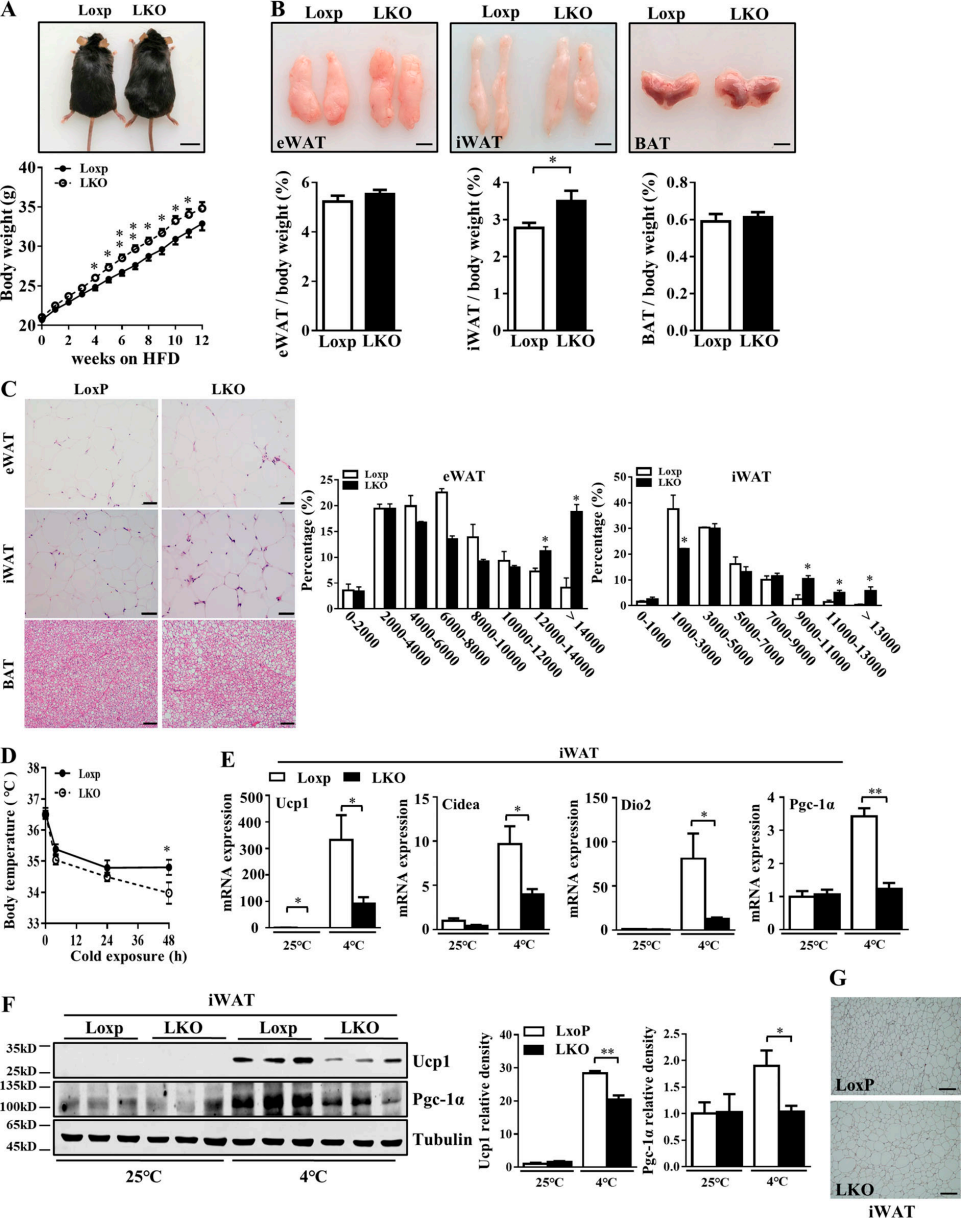
**LKO exacerbated HFD-induced obesity by impairing thermogenesis.** LoxP and LKO mice were fed with HFD for 12 wk. **(A)** Appearance (top) and growth curve (bottom) of LoxP (*n* = 29) and LKO (*n* = 26) mice after HFD. Scale bar = 2 cm. **(B)** Representative photographs of adipose tissues (top) and the ratio of fat depots to body weight (bottom) from LoxP (*n* = 14) and LKO (*n* = 13) mice. Scale bar = 1 cm. **(C)** Histological analysis of adipose tissues and distribution of adipocyte size of eWAT and iWAT (*n* = 8). **(D)** The rectal temperature of LoxP and LKO mice during cold exposure (4°C; *n* = 6). **(E)** mRNA levels of thermogenic genes in iWAT from Loxp and LKO mice (*n* = 6). **(F)** Protein levels of Ucp1 and Pgc-1α in iWAT from Loxp and LKO mice. **(G)** Representative images of Ucp1 IHC. Scale bar = 50 µm. Each experiment was independently performed two to three times. All data are mean ± SEM. *, P < 0.05; **, P < 0.01.

To further confirm the role of Manf in energy homoeostasis, we generated LKO mice (Fig. S4 A). WB analysis confirmed that Manf was specifically deleted in the liver (Fig. S4, B and C). Serum Manf level was also lower in LKO than Loxp mice (Fig. S4 D). Loxp and LKO mice on a CD showed indistinguishable body weight, indistinguishable body length, and similar weight and morphology of tissues (Fig. S4, E–H) and insulin sensitivity (Fig. S4 I). When challenged with an HFD, LKO mice were larger and gained more weight than control littermates (Fig. 6 A). In agreement with these findings, the iWAT weight was significantly increased in LKO mice (Fig. 6 B). In addition, the adipocyte cell size was larger in LKO iWAT than control iWAT (Fig. 6 C).

### Manf ablation impaired thermogenesis in iWAT and aggravated HFD-induced hepatic steatosis and inflammation in eWAT

**Figure S5.**
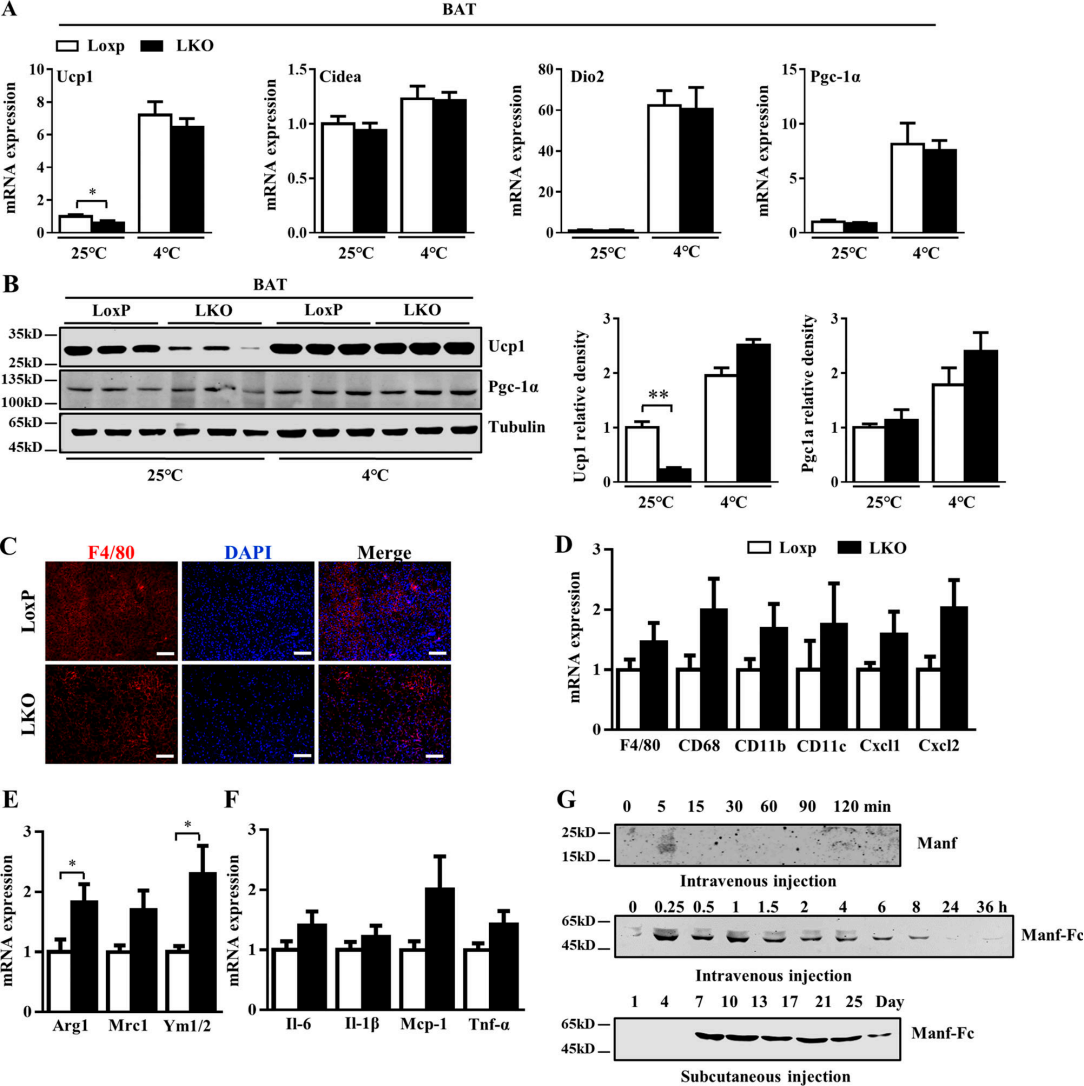
**The effect of LKO on thermogenesis and inflammation in BAT. (A)** qPCR analysis of thermogenic genes in BAT from mice under CD for 12 wk (*n* = 7 or 8). **(B)** The protein levels Ucp1 and Pgc-1α in BAT from mice fed with CD for 12 wk. **(C)** Immunofluorescent staining of F4/80 in BAT from HFD-fed mice. **(D–F)** mRNA levels of markers of M1 and M2 macrophages and inflammatory genes in BAT from HFD-fed LoxP (*n* = 7) and LKO mice (*n* = 6). **(G)** Pharmacodynamics of MANF and Manf-Fc. Serum was collected at the indicated times after single injection of the Manf or Manf-Fc in mice. Serum Manf level was detected by WB analysis. Each experiment was independently performed two or three times. All data are mean ± SEM. *, P < 0.05; **, P < 0.01.

We next explored whether LKO of Manf affected the thermogenesis of adipose tissue. LKO mice showed impaired cold tolerance (Fig. 6 D), which suggests that Manf ablation in liver weakened thermogenesis by decreasing the browning of iWAT. Moreover, KO of Manf in the liver greatly decreased the mRNA expression of thermogenic genes (Fig. 6 E). Consistently, the protein levels of Ucp1 and Pgc-1α were down-regulated in iWAT of LKO mice (Fig. 6, F and G). In BAT, the expression of thermogenic genes was not changed except for *Ucp1* at 25°C. This difference was diminished after cold exposure (Fig. S5, A and B).

**Figure 7.**
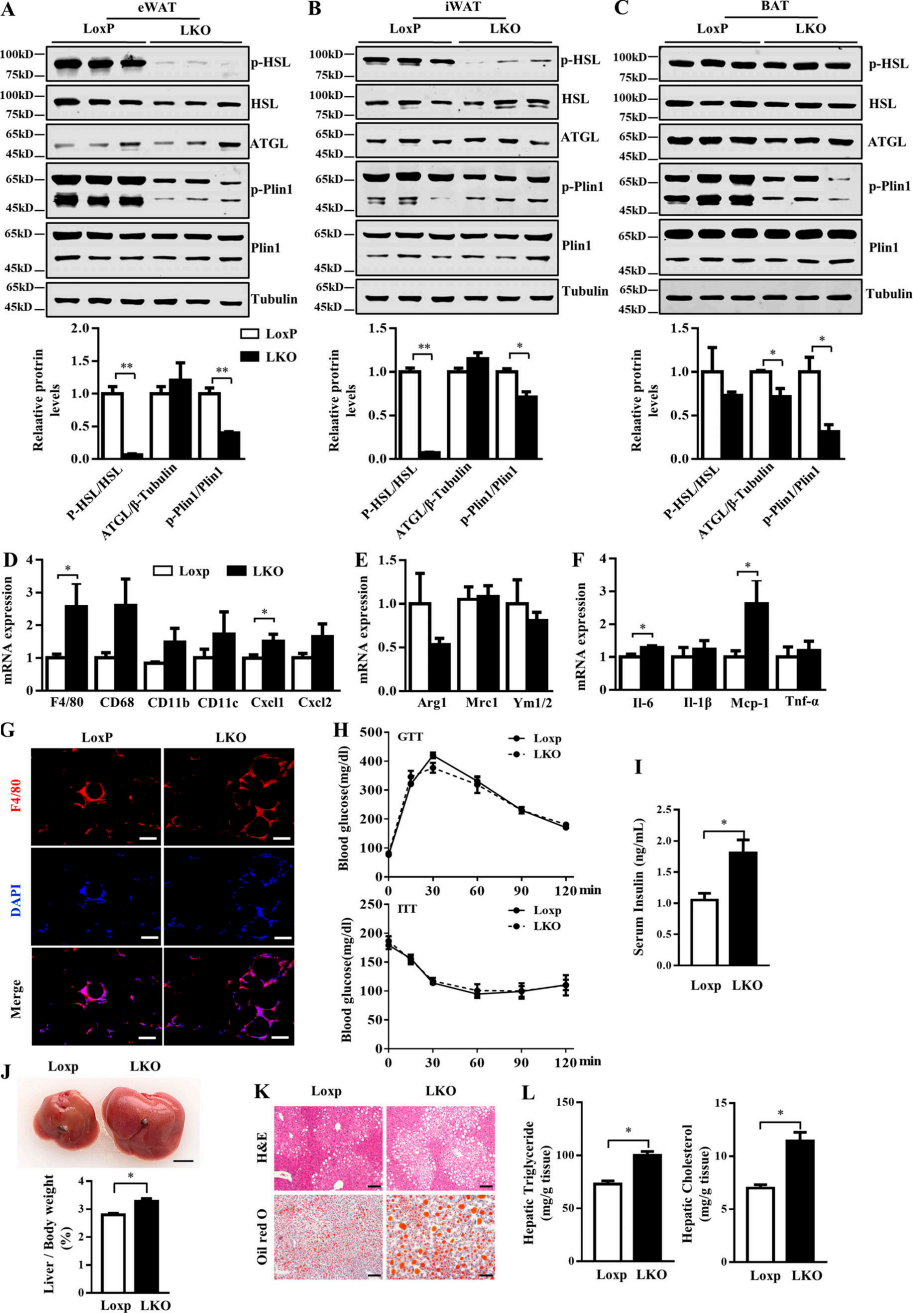
**Liver-specific Manf ablation inhibited lipolysis and aggravated HFD-induced hepatic steatosis. (A–C)** Protein levels of key lipolytic proteins in eWAT (A), iWAT (B), and BAT (C) from LoxP and LKO mice fed an HFD (*n* = 3). **(D–F)** mRNA levels of markers of M1 and M2 macrophages and inflammatory genes in eWAT from HFD-fed mice (*n* = 5 or 6). **(G)** Immunofluorescent staining of F4/80 in eWAT from HFD-fed mice. Samples were stained with F4/80 (red) or DAPI (blue). **(H)** Blood glucose levels during a GTT (1 g/kg) and an ITT (1.5 U/kg) performed on LoxP and LKO mice under HFD (*n* = 6). **(I)** Serum insulin levels in overnight-fasted LoxP and LKO mice (*n* = 6). **(J)** A representative image of liver and ratio of liver to body weight from LoxP and LKO mice (*n* = 13). Scale bar = 0.5 cm. **(K)** H&E and Oil Red O staining of liver sections. Scale bar = 50 µm. **(L)** Hepatic lipids levels in liver of LoxP and LKO mice fed an HFD (*n* = 6). Each experiment was independently performed two to three times. All data are mean ± SEM. *, P < 0.05; **, P < 0.01.

Lipolysis that coordinated with thermogenesis was decreased in LKO mice. The phosphorylation of HSL was significantly decreased in both eWAT and iWAT of LKO mice as compared with LoxP mice (Fig. 7, A and B). The phosphorylation of Plin1 was inhibited in eWAT, iWAT, and BAT of LKO mice (Fig. 7, A–C). In eWAT of LKO mice, the expression of M1 macrophage markers was increased (Fig. 7 D), whereas that of M2 macrophage markers was not changed (Fig. 7 E). The mRNA levels of IL-6 and Mcp-1 were increased in eWAT of LKO mice (Fig. 7 F). In addition, F4/80 staining showed greater macrophage infiltration in LKO than LoxP eWAT (Fig. 7 G). In BAT, the markers of M2 macrophage were slightly increased (Fig. S5, C–F), and the expression of inflammatory genes was not changed (Fig. S5 F). Glucose tolerance test (GTT) and insulin tolerance test (ITT) were barely changed in LKO mice fed an HFD (Fig. 7 H). However, serum insulin level was indeed induced in LKO mice (Fig. 7 I). Furthermore, the liver size was larger for LKO mice than controls (Fig. 7 J). LKO markedly exacerbated diet-induced steatosis in the liver, which was confirmed by Oil Red O staining and increased level of triglycerides in the liver (Fig. 7, K and L).

### Recombinant Manf improved metabolic disorders in obese mice

**Figure 8.**
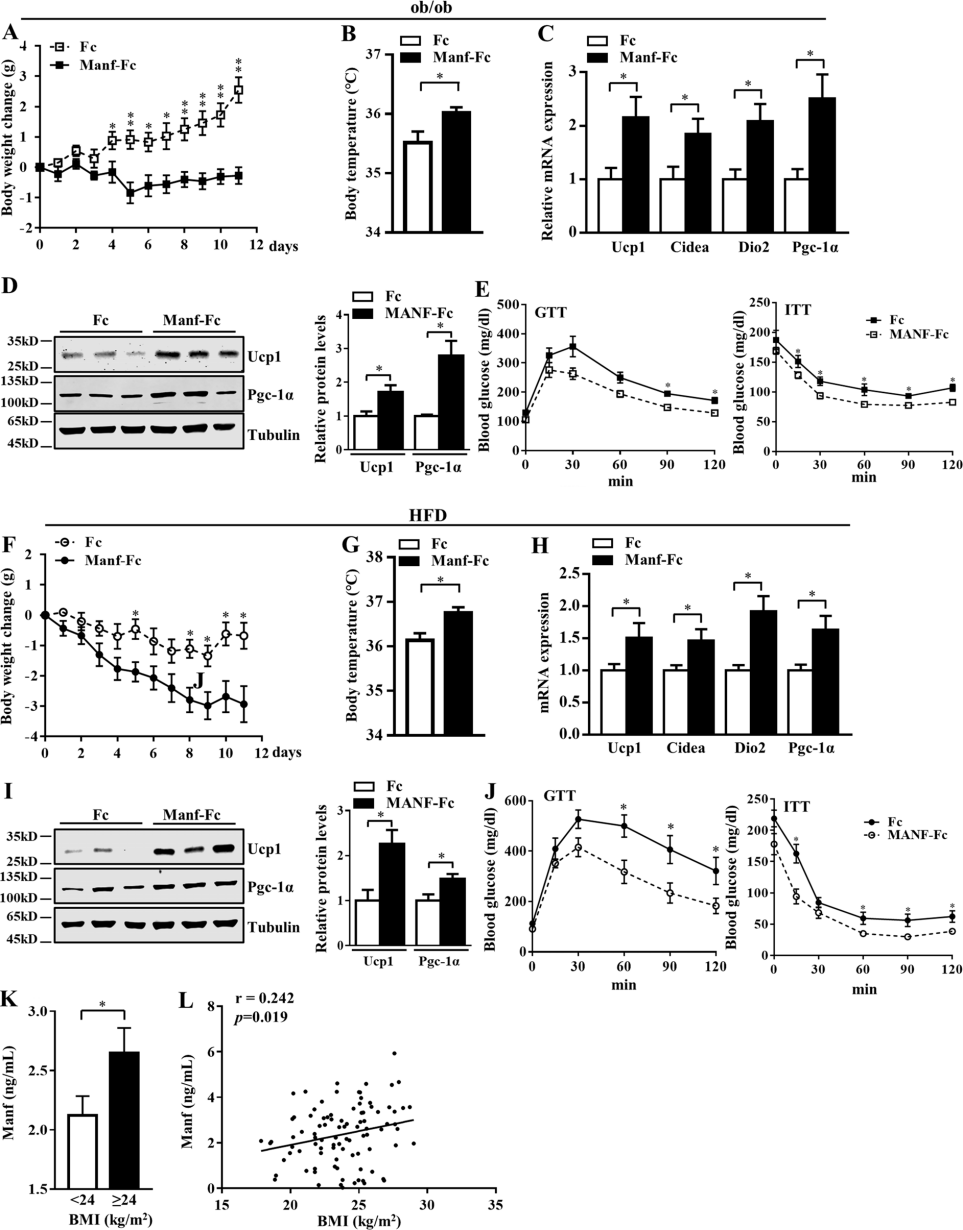
**Manf improved metabolic disorders in obese mice and positively correlated with BMI in human. (A–K)** Ob/ob and HFD-fed mice were treated with Fc or Manf-Fc weekly (0.3 mg/kg; *n* = 6). Body weight change (A and F) and rectal temperature (B and G) in HFD-fed mice treated with Fc or Manf-Fc. mRNA (C and H) and protein levels (D and I) of thermogenic genes in iWAT from treated mice. **(E and J)** Blood glucose concentrations during GTT (1 g/kg) and ITT (2 U/kg) in HFD-fed mice treated with Fc or Manf-Fc. **(K)** Comparison of Manf levels between groups. **(L)** Correlation between Manf and BMI. Each experiment was independently performed two to three times. All data are mean ± SEM. *, P < 0.05; **, P < 0.01.

To study the therapeutic potential of Manf, we fused Manf with the mouse IgG Fc fragment to generate a long-acting form of Manf (Manf-Fc). Ob/ob mice were subcutaneously injected weekly with Fc or Manf-Fc (0.3 mg/kg) according to Manf-Fc pharmacodynamics in the blood (Fig. S5 G). Manf-Fc–treated mice showed a marked loss of body weight as compared with control mice (Fig. 8 A). Consistent with results from Tg mice, Manf-Fc–treated mice showed higher body temperature (Fig. 8 B) and mRNA (Fig. 8 C) and protein levels (Fig. 8 D) of thermogenic genes in iWAT than Fc-treated mice. Manf-Fc treatment also improved glucose tolerance and insulin resistance (Fig. 8 E). In the diet-induced obesity model, similar results were observed. Manf-Fc reversed the gain of body weight (Fig. 8 F), increased body temperature (Fig. 8 G) and expression of thermogenic genes (Fig. 8, H and I), and improved the GTT and ITT results (Fig. 8 J) in obese mice.

### Manf was positively correlated with BMI in humans

To explore the relationship of Manf with obesity in humans, we measured circulating Manf level in 94 nondiabetic subjects. The characterization of these subjects is listed in Table 1. Manf level was higher in overweight and obese subjects (BMI ≥24) compared with nonobese humans (BMI <24; Fig. 8 K). Correlation analysis revealed that Manf was positively correlated with BMI (Fig. 8 L).

**Table 1.** Anthropometric characteristics and metabolic status of study subjects

	Normal (*n* = 50)	Overweight (*n* = 44)	P value
Age, yr	51.69 (47.75–60.20)	60.11 ± 9.48	0.006
Female	34 (68%)	25 (56.8%)	0.263
BMI, kg/m^2^	22.18 (20.56–23.32)	25.54 (25.06–27.18)	<0.001
W/H ratio	0.86 ± 0.59	0.89 (0.87–0.93)	0.005
Hb, g/liter	137.50 (131.75–151.00)	137.09 ± 18.38	0.710
HbA_1C_, %	5.20 (4.95–5.40)	5.25 ± 0.44	0.141
I_0_, mIU/liter	6.28 ± 3.15	7.12 (5.34–9.40)	0.079
I_30_, mIU/liter	50.10 (30.28–76.71)	56.50 (39.00–82.21)	0.168
I_120_, mIU/liter	37.50 (18.77–58.81)	47.22 ± 29.54	0.386
FPG, mM	4.65 (4.40–4.90)	4.75 ± 0.38	0.185
G_30_, mM	7.95 (6.67–8.60)	8.83 ± 2.06	0.011
G_120_, mM	5.47 ± 1.07	5.67 ± 1.04	0.356
TG, mM	1.05 (0.70–1.50)	1.40 (1.12–2.07)	0.001
TC, mM	4.68 ± 0.92	4.86 ± 0.82	0.331
HDL-C, mM	1.60 (1.30–1.90)	1.40 (1.20–1.50)	0.003
LDL-C, mM	2.72 ± 0.64	2.87 ± 0.73	0.244
ALT, U/liter	20.00 (16.00–27.50)	24.00 (17.25–32.00)	0.187
AST, U/liter	22.00 (19.75–26.25)	26.95 ± 8.58	0.026

Kolmogorov-Smirnov test was used to test continuous variables for normal distribution. Data are mean ± SD, median (interquartile range), or number (%). Continuous data with normal distribution were analyzed by two-tailed Student *t* test. Nonnormally distributed continuous data were analyzed by Mann–Whitney *U* test. Categorical data are shown as frequency (%) and were analyzed by χ^2^ test. ALT, alanine aminotransferase; AST, aspartate aminotransferase; FPG, fasting plasma glucose; Hb, hemoglobin; HbA_1C_, glycosylated hemoglobin; HDL-C, high-density lipoprotein cholesterol; LDL-C, low-density lipoprotein–cholesterol; mIU, milli–international units; TC, total cholesterol; TG, triglycerides; W/H, waist-to-hip ratio; I_0_, blood insulin level after over night fasting; I_30_ and I_120_, blood insulin level at 30 or 120 min point after oral glucose tolerance test; G_30_ and G_120_, blood insulin level at 30 or 120 min point after oral glucose tolerance test.

### Adipose Manf is dispensable for energy homeostasis

**Figure 9.**
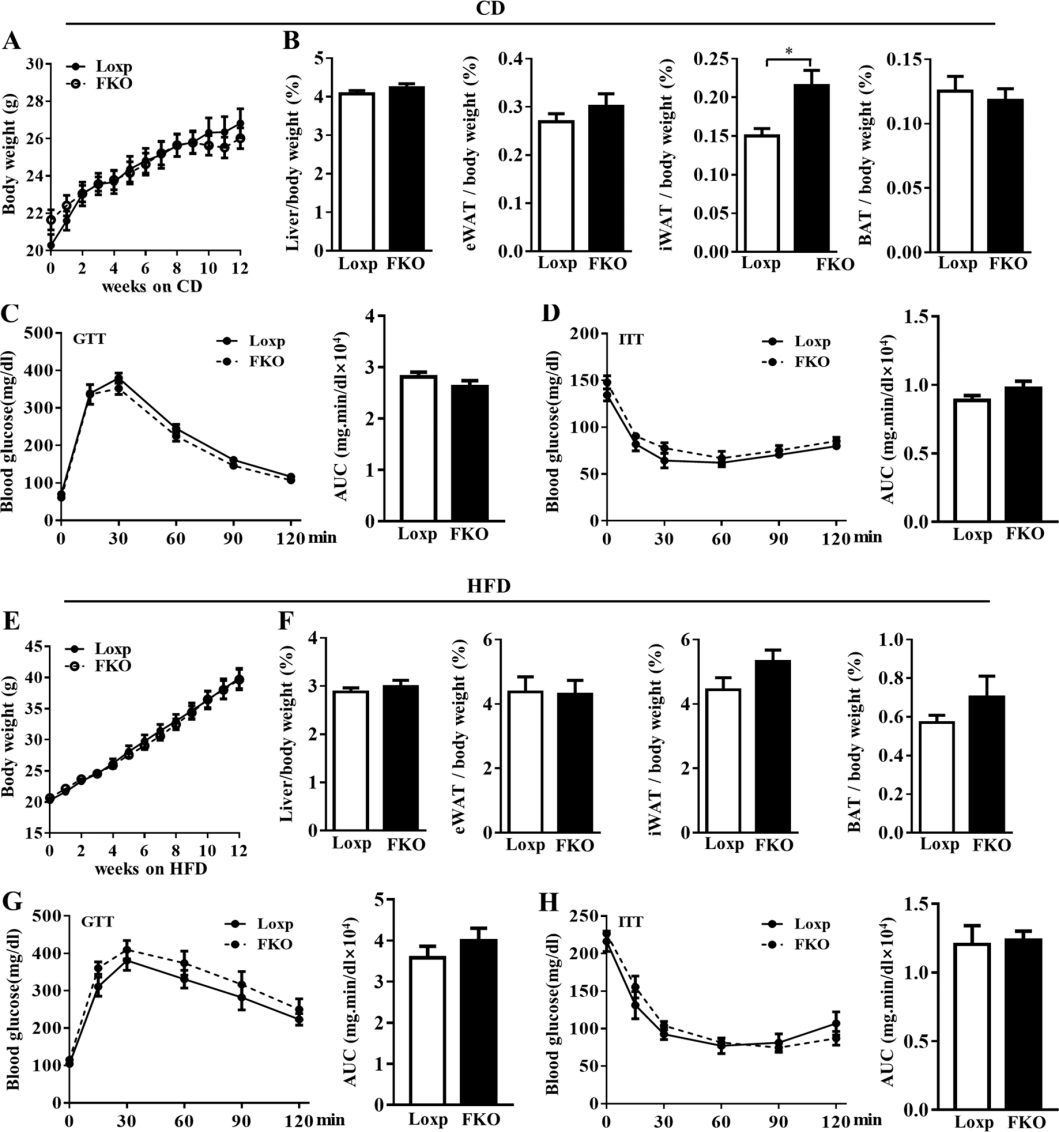
**FKO did not affect energy homeostasis.** LoxP and FKO mice were fed CD or HFD for 12 wk. **(A)** Growth curve of LoxP and FKO mice under CD (*n* = 6). **(B)** The ratio of liver, eWAT, iWAT, and BAT weight to body weight of mice under CD (*n* = 6). **(C and D)** Blood glucose levels during a GTT (2 g/kg; C) and an ITT (0.5 U/kg; D) under CD (*n* = 6). **(E)** Growth curve of LoxP and FKO mice under HFD (*n* = 9). **(F)** The ratio of liver, eWAT, iWAT, and BAT weight to body weight of mice under HFD (*n* = 9). **(G and H)** Blood glucose levels during a GTT (1 g/kg; G) and an ITT (1.5 U/kg; H) under HFD (*n* = 7–9). Each experiment was independently performed two to three times. All data are mean ± SEM. *, P < 0.05. AUC, area under the curve; IRES, internal ribosome entry site.

Manf is detectable in adipose tissue as well (Fig. 1 D). To test whether adipocyte-secreted Manf may function in an autocrine/paracrine manner, fat-specific Manf KO (FKO) mice were generated. However, under both the CD and HFD, FKO mice shared comparable body weight and tissue weights with LoxP mice (Fig. 9, A, B, E, and F). Also, GTT and ITT results did not differ between LoxP and FKO mice under both the CD (Fig. 9, C and D) and HFD (Fig. 9, G and H). These results suggest that adipose-secreted Manf is dispensable for whole-body energy homeostasis.

## Discussion

In this study, we revealed a novel function for Manf as a hepatokine in promoting thermogenesis and iWAT browning. Manf was highly expressed in the liver and regulated by different nutritional conditions. Liver-specific Manf overexpression markedly protected mice against diet-induced obesity and insulin resistance. The anti-obesity effect of Manf may result from the increased energy expenditure via browning of iWAT but not BAT. Further study indicated that Manf-regulated browning was mediated by p38 MAPK signaling. Conversely, LKO impaired the iWAT browning and exacerbated diet-induced obesity. Furthermore, recombinant Manf showed therapeutic effects by promoting thermogenesis in iWAT and improving insulin resistance in both diet-induced and genetic obese mouse models. Finally, circulating Manf level was positively correlated with BMI in humans.

Manf is known as an ERS–up-regulated protein (Apostolou et al., 2008; Mizobuchi et al., 2007). However, whether Manf is regulated by nutritional status is less studied. Yang et al. (2017) reported that Manf expression in the mouse hypothalamus was closely linked to feeding status. Both Manf mRNA and protein levels were up-regulated in response to fasting in the hypothalamus but not the liver. We discovered that 24-h fasting decreased Manf expression in mice, and 4-h refeeding increased Manf expression in the liver. This discrepancy may be due to the difference in fasting time, which was 48 h in the study by Yang et al. (2017) and 24 h in ours. Furthermore, future studies are necessary to reveal the signaling pathway regulating Manf expression.

Beige adipocyte is closely related to the ability to resist body weight gain and fat accumulation. Recruitment and activation of beige fat are controlled by several molecules and signaling pathways. p38 MAPK is a necessary component downstream of cAMP/protein kinase A signaling in brown adipocytes (Bordicchia et al., 2012). It phosphorylates and thereby activates ATF2 as well as Pgc-1α. Pgc-1α is an important coregulator in thermogenesis and can elicit the transcription of Ucp1 (Puigserver and Spiegelman, 2003). Accumulating studies showed that p38 MAPK signaling is required for browning (Ng et al., 2017; Zhang et al., 2014). Our data indicated that Manf directly stimulated the p-p38 and induced the expression of Pgc-1α and Ucp1. Blocking the p38 MAPK pathway by its inhibitor abolished Manf-induced p38 phosphorylation and decreased the expression of thermogenic genes. These results suggest that Manf promotes browning in iWAT, at least in part by activating p38 MAPK. Furthermore, thermogenesis demands fatty acid release from both eWAT and iWAT (Li et al., 2014). We found increased expression of ATGL and phosphorylation of HSL in both eWAT and iWAT. Consistently, the phosphorylation of Plin1 was higher. These results suggest that increased lipolysis may coordinate with thermogenesis and could explain the decreased mass weight of eWAT and iWAT.

Hepatokines are a class of proteins secreted by hepatocytes and can affect metabolic processes via autocrine, paracrine, or endocrine signaling. Manf was reported to be secreted into the extracellular space via the classic ER Golgi pathway and affect other cells (Apostolou et al., 2008). We found elevated levels of Manf in liver and serum after refeeding, and it could promote iWAT browning, which suggests that Manf may take part in diet-induced thermogenesis. Fgf21, another well-known hepatokine, is also an important thermogenic regulator but showed an opposite response to fasting as compared with Manf in the liver (Hondares et al., 2010). The hepatic and circulating levels of Fgf21 in mice are markedly up-regulated upon fasting and are suppressed by refeeding (Inagaki et al., 2007). Fgf21 can also be detected in adipose tissue. In contrast to liver, adipose Fgf21 is low during fasting but is greatly induced after feeding (Dutchak et al., 2012). More importantly, the secreted Fgf21 from WAT acts locally, but does not enter the circulation (Dutchak et al., 2012). Adipose-released Fgf21 promoted thermogenesis through an autocrine and paracrine manner. Therefore, adipose paracrine and autocrine Fgf21 and hepatic Manf coregulate browning and may play a role in diet-induced thermogenesis.

Fgf21 acts on adipose tissues via the adipose-enriched coreceptor β-klotho and FGF receptor 1c (Foltz et al., 2012); the receptor of Manf on the cell membrane has yet to be identified. According to clues from the structure, the N-terminal domain of Manf is a saposin-like domain. Saposins can interact with membranes, so Manf may directly bind to membrane lipids. Also, the C terminus of Manf contains an RTDL (Arg-Thr-Asp-Leu) sequence. These highly conserved final four amino acids resemble the KDEL (Lys-Asp-Glu-Leu) sequence, which is a canonical ER retention signal. The peptide that can interact with KDEL receptors inhibited the binding of Manf to the plasma membrane, which suggests that Manf probably binds to the cell surface by interacting with the KDEL receptors (Henderson et al., 2013). Furthermore, KDEL receptors have been shown to modulate the activity of p38 MAPK (Yamamoto et al., 2003). Future studies are needed to confirm and characterize the existence of a Manf-specific receptor on the membrane of adipose tissues.

Serum levels of Manf were increased in obese mice and overweight humans. In addition, correlation analysis demonstrated a positive association of serum Manf level with BMI. We reasoned that the higher levels of serum Manf in obese mice and humans may be the result of its compensatory up-regulation, like the hyperinsulinemia and high level of leptin in obese subjects. However, pharmacological administration of Manf showed a promising therapeutic effect on adiposity, hyperglycemia, and hyperinsulinemia in diet-induced and genetic obese mouse models. We speculated that endogenous Manf is not sufficient, but a supraphysiological dose of Manf could combat obesity. Further studies are still needed to explain the contradiction and explore the underlying mechanism of increased Manf in human obesity. Taken together, the present results uncovered a role for Manf as a hepatokine in promoting energy metabolism by activating browning of iWAT. Manf may be a novel target for obesity and related metabolic disorders.

## Materials and methods

### Animals

All animal procedures were approved by the Animal Care and Use Committee of Sichuan University. Mice were housed at 25°C in a 12-h light–dark cycle in the animal facility at West China Hospital, Sichuan University. Food and water were available ad libitum. Tg mice on a C57BL/6J background were created by using a control albumin promoter. Homozygous transgenic (Tg) mice and WT littermates were used. Albumin-Cre and adiponectin-Cre mice on a C57BL/6J background were purchased from The Jackson Laboratory. Manf^Loxp/Loxp^ (Loxp) mice on a C57BL/6J background were created by inserting Loxp sites flanking exons 2 of Manf. Loxp mice were crossed with albumin-Cre and adiponectin-Cre animals to generate LKO mice and FKO mice, respectively. Loxp littermates were used as controls. For diet-induced obesity, transgenic, KO, and control male mice were fed an HFD (D12492; Research Diet) for 12 wk. For cold exposure, mice were housed at 4°C with adequate food and water for 72 h. Body temperature was monitored by using a rectal probe. For therapeutic studies of Manf, ob/ob and HFD-induced obese WT mice received a subcutaneous injection of Fc or Manf-Fc (0.3 mg/kg body weight) weekly. Ob/ob and C57BL/6J WT mice were purchased from Beijing HFK Bioscience.

### GTT and ITT

For GTT, mice were fasted for 16 h before receiving an intraperitoneal injection of glucose at the indicated dosage. For ITT, mice were fasted for 4 h before receiving an intraperitoneal injection of human insulin (Novolin) at the indicated dosage. Blood glucose was measured at the indicated time points by a glucose meter.

### Biochemical analysis

Serum concentrations of triglycerides, cholesterol, and glucose were measured by using kits from Zhongsheng Technologies. Serum levels of insulin and leptin were measured by using ELISA kits from Cayman. To analyze hepatic lipids, 150 mg liver tissue was homogenized in 1 ml PBS. Homogenates were extracted with chloroform/methanol (2:1, vol/vol) and dissolved in 1% Triton X-100 in ethanol. Hepatic triglycerides and cholesterol levels were measured by using the reagents described above and normalized to liver weight.

### Flow cytometry

To detect macrophage infiltration, stromal vascular cells (SVCs) from epididymal WAT were prepared and analyzed. Epididymal fat pads from mice fed an HFD were washed, minced, and digested with type II collagenase (Sigma-Aldrich) for 30 min at 37°C. The cell suspension was filtered with a 100-µm cell strainer, then centrifuged at 500 *g* for 5 min at 4°C to separate adipocytes from the SVC pellet. The SVC pellet was then disrupted and incubated with 0.5 ml RBC lysis buffer (155 mM NH_4_Cl, 10 mM KHCO_3_, and 0.1 M EDTA) at room temperature for 5 min. After centrifugation at 500 *g* for 5 min at 4°C, SVCs were resuspended in PBS containing 1 mM EDTA, 25 mM Hepes, and 1% FBS and incubated with Fc-block (anti-CD16/32) on ice for 10 min, then with a combination of the following fluorescence-labeled antibodies at 4°C for 30 min: anti-F4/80 (PE; 0.2 µg/10^6^ cells; 12–4801; Affymetrix), CD11c (APC; 0.2 µg/10^6^ cells; 561119; BD Biosciences), CD11b (FITC; 0.5 µg/10^6^ cells; 561688; BD Biosciences), and CD206 (Cy7; 0.2 µg/10^6^ cells; 141719; Biolegend). Cells were analyzed by using CytoFlex (Beckman).

### Histological analysis

For H&E staining, tissues were fixed in 10% formalin, dehydrated, embedded in paraffin, sectioned at 5 µm, and stained with H&E. For IHC and IF, sections of tissues were deparaffinized and rehydrated, and underwent antigen retrieval byzusing heat-induced epitope methods. Frozen liver sections (8 µm) were used for Oil Red O staining. The images were captured by a light microscope (Nikon). The size of adipocyte cells was analyzed by using National Institutes of Health ImageJ software.

### [^18^F]-FDG micro-PET/CT

Mice were fasted overnight, anesthetized using 2% isofluorane in oxygen, and tail vein–injected with ∼50 µCi of [^18^F]-FDG. The animals were replaced in the cage and permitted to roam freely for [^18^F]-FDG uptake. The micro-PET/CT imaging started ∼30 min after the [^18^F]-FDG injection. Mice were placed on the micro-CT pallet (animal bed) under 2% isofluorane anesthesia for imaging. Mice were sacrificed after PET/CT imaging, and tissues were collected and measured by using a γ counter. All experiments took place at room temperature.

### Running distance analysis

Running distance was measured using an automatic recording and rhythm analysis system of small animal spontaneous activity. Mice were separately kept in the cages with a running wheel (17 cm diameter) connected to the computer. Cages were put into the compartments with a light control system (07:00–19:00 light, 19:00–07:00 dark). After three-dimensional domestication, data were collected by an ACT-500 CLOCKLAB Rhythm Biological Data Acquisition and Analysis System for 6 d.

### Isolation and differentiation of primary beige adipocytes and brown adipocytes

The interscapular brown fat pad was isolated from newborn mice, minced, then digested for 30 min at 37°C in isolation buffer with 0.75 mg/ml type II collagenase (Sigma-Aldrich). The cell suspension was filtered with a 100-µm cell strainer, then centrifuged for 10 min at 1,500 rpm to pellet the SVF cells. SVF cells were seeded in collagen-coated 24-well plates with complete culture medium (DMEM containing 10% FBS). For preadipocyte differentiation, confluent cells were incubated with induction medium (DMEM containing 10% FBS, 1 µg/ml insulin, 0.5 μM isobutylmethylxanthine, 10 µM pioglitazone, 1 μM dexamethasone, and 1 nM triiodothyronine). After 48 h, the medium was replaced with maintenance medium (DMEM supplemented with 10% FBS, 1 µg/ml insulin, and 1 nM triiodothyronine). Fresh maintenance medium was added every 2 d.

Primary subcutaneous white adipose SVFs were isolated and differentiated. Briefly, subcutaneous fat pads from 6-wk-old WT mice were minced and digested with type II collagenase for 20 min at 37°C. Digestion was stopped and was followed by centrifugation at 700 *g* for 10 min. After resuspension, cells were filtered through a 100-µm strainer and centrifuged again. SVF cells were then resuspended, seeded in collagen-coated 24-well plates, and cultured with F12 containing 10% FBS. Adipocyte differentiation was induced by culturing confluent cells in medium containing 10% FBS, 5 µg/ml insulin, 0.5 mM isobutylmethylxanthine, 1 µM dexamethasone, 1 nM triiodothyronine, and 10 µM pioglitazone. After 48 h, cells were maintained in medium containing 10% FBS, 1 nM triiodothyronine, and 5 µg/ml insulin until harvesting. Fresh maintenance medium was added every 2 d. In some experiments, differentiated adipocytes were treated with CM collected from primary hepatocytes, recombinant human Manf (PeproTech), or recombinant human FGF21 (PeproTech) as indicated.

### Isolation, culture, and treatment of mouse primary hepatocytes

6- to 8-wk-old male WT mice were anesthetized with ketamine and xylazine. The inferior vena cava was cannulated, and the liver was perfused with 50 ml prewarmed 37°C Hank’s buffer containing 50 µM EGTA and 50 mM Hepes, pH 7.4, then isolated, and the liver capsule was removed. The entire liver was digested for 10 min at 37°C with 1 mg/ml type I collagenase (Sigma-Aldrich). The cell suspension was filtered through a 100-µm strainer, centrifuged at 50 *g* for 3 min, and washed three times with DMEM. Resuspended hepatocytes were seeded at 2 × 10^5^ cells per well in collagen-coated 6-well plates. Cells were cultured with DMEM containing 10% FBS at 37°C with 5% CO_2_. To collect CM, the medium was replaced 2 h after infection with Ad-GFP or Ad-Manf. The CM was collected 24 h after Ad infection and stored at −80°C.

### Oxygen consumption rate of primary brown adipocytes

Oxygen consumption rate in brown adipocytes was analyzed by the Seahorse XF Cell Mito stress test kit (Agilent Technologies). Primary brown adipocytes were isolated and differentiated in Seahorse XF Cell Culture Miniplate. Adipocytes were incubated with recombinant human MANF for 48 h. Before the assay, maintenance medium was replaced with pH-adjusted seahorse base medium (10 mM glucose, 1 mM pyruvate, and 2 mM glutamine), and then cells were incubated at 37°C in a non-CO_2_ incubator for 1 h. Oligomycin (2 µM), carbonyl cyanide-4 (trifluoromethoxy) phenylhydrazone (1 µM), and antimycin A (1 µM) were sequentially loaded into the hydrated sensor cartridge. All the data were analyzed with the software Wave (Agilent Technologies).

### Fatty acid oxidation

Fatty acid oxidation was determined by conversion of ^13^C-labeled mixed fatty acids into intermediate products of tricarboxylic acid cycle. Differentiated primary BAT were pretreated with Manf for 48 h, and then incubated with ^13^C-labeled mixed fatty acids (0.4 mM, palmitic acid 41.6%, palmitoleic acid 6.5%, stearic acid 2.5%, oleic acid 31.1%, linoleic acid 14.5%, and linolenic acid 3.8%) or fatty acid–free BSA (1.2%) for 1 h at 37°C. Cells were washed with 1 × PBS twice, and 1 ml precooling methanol/water (8:2) with 2 μl 0.75 mg/ml myristic acid d27 was added into cells. The cells were collected into 1.5-ml microtube, then stored at −80°C for 30 min, vortexed at 4°C for 3 min, and subjected to ultrasound in an ice bath for 15 min. After being centrifuged for 10 min (13,000 rpm, 4°C), 800 µl supernatant was isolated, and another 400 µl methanol/water (8:2) was added into precipitation, vortexed at 4°C for 3 min, and centrifuged for 10 min (13,000 rpm, 4°C). Isolated 400 µl supernatant was added into the same microtube with the previous step. After being vacuum concentrated at 30°C for 3 h, 30 μl methoxide hydrochloride-pyridine solution (40 mg/ml) was added and reacted at 60°C for 90 min. Then, N-methyl-N-(trimethylsilyl)trifluoroacetamide was added and reacted at 60°C for 90 min. After centrifugation, supernatant was used for gas chromatography mass spectrometry/mass spectrometry analysis.

### WB

Tissues and cultured cells were homogenized in lysis buffer. Lysates were moved at 12,000 rpm for 10 min. Equal amounts of protein were loaded on SDS-PAGE gels and probed with antibodies. Blots were then detected by the LI-COR Odyssey System. To prepare serum samples for immunoblotting, 60 µl of serum from mice was precleared for albumin/IgG by using the kit from Millipore as recommended by the manufacturer. Samples were then mixed with 6 × SDS loading buffer. The mixture was boiled for 5 min and analyzed by using WB. The following antibodies were used: rabbit-anti-MANF (1:10,000 for WB; SAB3500384; Sigma-Aldrich), rabbit-anti-Ucp1 (1:4,000 for WB, 1:400 for IHC; Ab10983; Abcam), rat-anti-F4/80 (1:200 for IF; Ab6640; Abcam), mouse-anti-Pgc-1α (1:2,000 for WB; Sc-517380; Santa Cruz Biotechnology), rabbit-anti-p-Akt (1:2,000 for WB; Ser 473; Sc-7985-R; Santa Cruz Biotechnology), rabbit-anti-Akt (1:2,000 for WB; Sc-8312; Santa Cruz Biotechnology), rabbit-anti-HSL (1:2,000 for WB; Sc-25843; Santa Cruz Biotechnology), mouse-anti-p-p38 (1:2,000 for WB; Sc-166182; Santa Cruz Biotechnology), rabbit-anti-p-HSL (1:2,000 for WB; 4126; Cell Signaling Technology), rabbit-anti-ATGL (1:2,000 for WB; 2138s; Cell Signaling Technology), rabbit-anti-p-Plin1 (1:2,000 for WB; 100G7E; Cell Signaling Technology), rabbit-anti-Plin1 (1:2,000 for WB; 9349; Cell Signaling Technology) rabbit-anti-p38 (1:2,000 for WB; BM4142, Boster), rabbit-anti-ATF2 (1:2,000 for WB; BA0653; Boster), rabbit-anti-p-ATF2 (1:2,000 for WB; AP1051; Abclonal), rabbit-anti-β-actin (1:100,000 for WB; AC026; Abclonal), and mouse-anti-β-tubulin (1:4,000 for WB; 200608; Zen Bio Science).

### RT-PCR and microarray assay

RNA was extracted from cells or frozen tissues by using Trizol reagent (Invitrogen). RT of 1 µg RNA involved use of an RT kit (Takara). SYBR Green–based RT-PCR was performed with the CFX96 Real-Time System (Bio-Rad). The following primers were used: *Arg1* forward 5′-AGA​CCA​CAG​TCT​GGC​AGT​TG-3′, *Arg1* reverse 5′-CCA​CCC​AAA​TGA​CAC​ATA​GG-3′; *Cidea* forward 5′-TCC​TAT​GCT​GCA​CAG​ATG​ACG-3′, *Cidea* reverse 5′-TGC​TCT​TCT​GTA​TCG​CCC​AGT-3′; *CD68* forward 5′-CAA​GGG​GGC​TCT​TGG​GAA​CTA-3′, *CD68* reverse 5′-GCT​CTG​ATG​TAG​GTC​CTG​TTT​G-3′; *CD11b* forward 5′-TTT​TAG​GAG​CAC​CTC​GGT​AT-3′, *CD11b* reverse 5′-TGA​GGA​TCA​AGT​TGG​TAT​TG-3′; *CD11c* forward 5′-AAA​ATC​TCC​AAC​CCA​TGC​TG-3′, *CD11c* reverse 5′-CAC​CAC​CAG​GGT​CTT​CAA​GT-3′; *Cxcl1* forward 5′-CTG​GGA​TTC​ACC​TCA​AGA​ACA​TC-3′, *Cxcl1* reverse 5′-CAG​GGT​CAA​GGC​AAG​CCT​C-3′; *Cxcl2* forward 5′-CCA​ACC​ACC​AGG​CTA​CAG​G-3′, *Cxcl2* reverse 5′-GCG​TCA​CAC​TCA​AGC​TCT​G-3′; *Dio2* forward 5′-CAT​TGA​TGA​GGC​TCA​CCC​TTC-3′, *Dio2* reverse 5′-GGT​TCC​GGT​GCT​TCT​TAA​CCT-3′; *F4/80* forward 5′-CAG​TCA​GAT​GAT​TCA​GAC​GGA​GT-3′, *F4/80* reverse 5′-GTC​ACA​GTG​CCA​CCA​ACA​AC-3′; *Fabp4* forward 5′-GTG​TGA​TGC​CTT​TGT​GGG​AAC-3′, *Fabp4* reverse 5′-CAT​GCC​TGC​CAC​TTT​CCT​TGT-3′; *IL-6* forward 5′-CTG​CAA​GAG​ACT​TCC​ATC​CAG-3′, *IL-6* reverse 5′-AGT​GGT​ATA​GAC​AGG​TCT​GTT​GG-3′; *IL-1β* forward 5′-GCA​ACT​GTT​CCT​GAA​CTC​AAC​T-3′, *IL-1β* reverse 5′-ATC​TTT​TGG​GGT​CCG​TCA​ACT-3′; *Mcp-1* forward 5′-GAG​GAC​AGA​TGT​GGT​GGG​TTT-3′, *Mcp-1* reverse 5′-AGG​AGT​CAA​CTC​AGC​TTT​CTC​TT-3′; *Mrc1* forward 5′-TGG​ATG​GAT​GGG​AGC​AAA​GT-3′, *Mrc1* reverse 5′-GCT​GCT​GTT​ATG​TCT​CTG​GC-3′; *Manf* forward 5′-TGC​TGC​CAC​CAA​GAT​CAT​CAA-3′, *Manf* reverse 5′-AGG​TCC​ACT​GTG​CTC​AGG​TCA​A-3′; *Pgc1α* forward 5′-GCA​CCA​GAA​AAC​AGC​TCC​AAG-3′, *Pgc1α* reverse 5′-CGT​CAA​ACA​CAG​CTT​GAC​AGC-3′; *Tnf-α* forward 5′-CAG​GCG​GTG​CCT​ATG​TCT​C-3′, *Tnf-α* reverse 5′-CGA​TCA​CCC​CGA​AGT​TCA​GTA​G-3′; *Ucp1* forward 5′-TCT​CAG​CCG​GCT​TAA​TGA​CTG-3′, *Ucp1* reverse 5′-GGC​TTG​CAT​TCT​GAC​CTT​CAC-3′; *Ym1/2* forward 5′-TTA​TCC​TGA​GTG​ACC​CTT​CTA​AG-3′, *Ym1/2* reverse 5′-TCA​TTA​CCC​AGA​TAG​GCA​TAG​G-3′; and *18s* forward 5′-TTG​ACT​CAA​CAC​GGG​AAA​CC-3′, *18s* reverse 5′-AGA​CAA​ATC​GCT​CCA​CCA​AC-3′. For microarray assays, total liver RNA from fasted and refed mice was extracted and analyzed by Kangcheng Bio-Tech Inc.

### Human samples

Participants were recruited from communities of Chengdu, China. Patients with diabetes were excluded. Serum MANF level was measured by ELISA kits (cat. SEC300Hu; Uscn; Life Science, Inc.). The study was approved by the Biological Sciences Ethical Committee of West China Hospital of Sichuan University, China.

### Statistical analysis

Data are expressed as mean ± SEM. Statistical comparisons involved two-tailed Student *t* test (for comparing two experimental conditions) or one-way ANOVA multiple comparison test. Correlation of serum MANF level with BMI was tested by Pearson correlation analysis. P < 0.05 was considered statistically significant.

### Online supplemental material

Fig. S1 displays additional data related to Fig. 1, showing that liver-specific overexpression of Manf does not affect body weight, body length, and tissue morphology under CD. Fig. S2 displays additional data related to Fig. 2, showing that physical activity and the function of BAT are unchanged after overexpression of Manf. Fig. S3 displays additional data related to Figs. 3 and 4, showing that Manf has no influence on traditional pathway of browning and inflammation in BAT. Fig. S4 displays additional data related to Fig. 6, showing that LKO do not affect body weight, body length, tissue morphology, and glucose tolerance under CD. Fig. S5 displays additional data related to Figs. 6 and 7, showing that LKO does not impact thermogenesis and inflammation in BAT.
